# Genome-Wide Analysis of the Glutathione S-Transferase (GST) Genes and Functional Identification of *MdGSTU12* Reveals the Involvement in the Regulation of Anthocyanin Accumulation in Apple

**DOI:** 10.3390/genes12111733

**Published:** 2021-10-29

**Authors:** Yu-Wen Zhao, Chu-Kun Wang, Xiao-Yu Huang, Da-Gang Hu

**Affiliations:** National Key Laboratory of Crop Biology, Shandong Collaborative Innovation Center of Fruit & Vegetable Quality and Efficient Production, College of Horticulture Science and Engineering, Shandong Agricultural University, Tai’an 271018, China; zhaoyw9612@163.com (Y.-W.Z.); emilywtr@163.com (C.-K.W.); HXYhxySDAU@163.com (X.-Y.H.)

**Keywords:** apple, anthocyanin, glutathione S-transferase (GST), gene family, *MdGSTU12*

## Abstract

Anthocyanins have essential biological functions, affecting the development of horticultural production. They are synthesized in the cytoplasm through flavonoid metabolic pathways and finally transported into vacuoles for storage. Plant glutathione S-transferases (GSTs) are multifunctional enzymes involved in anthocyanin transportation. In this study, we identified 38 GSTs from the apple (*Malus domestica*) genome (HFTH1 Whole Genome v1.0) based on the sequence similarity with the GST family proteins of *Arabidopsis*. These *MdGST* genes could be grouped into nine chief subclasses: U, F, L, Z, T, GHR, EF1Bγ, TCHQD, and DHAR. The structures, motifs, three-dimensional models, and chromosomal distribution of *MdGST* genes were further analyzed. Elements which are responsive for some hormones and stress, and others that involve genes related to flavonoid biosynthesis were forecast in the promoter of *MdGST*. In addition, we identified 32 orthologous gene pairs between apple and *Arabidopsis*. These genes indicated that numerous apple and *Arabidopsis* counterparts appeared to be derived from a common ancestor. Amongst the 38 *MdGST* genes, *MdGSTU12* was considerably correlated with anthocyanin variation in terms of extracting expression profiles from reported. Finally, further functional identification in apple transgenic calli and subcellular localization confirmed that *MdGSTU12* was of great significance in anthocyanin accumulation in apple.

## 1. Introduction

Flavonoids almost exist in all higher plants in nature as a secondary metabolite and they have various biological functions. For example, they are the main regulators of plant auxin transport and can also be used as a protective agent for plants to cope with biotic and abiotic stresses [1]. Anthocyanin is a kind of water-soluble pigment in flavonoid compounds, which is widely distributed in various plants in nature. The expression of this color depends, to a certain extent, on the PH value of vacuoles [2,3].

The existence of anthocyanin not only makes nature more colorful but also has economic significance. Recently, many breeding and improvement programs of horticultural crops have documented the modification of anthocyanin-related components as the primary purpose [3]. Currently, there are many studies on the anthocyanin biosynthetic pathways in horticultural and agricultural crops. Previous studies have also showed that anthocyanin synthesis played an important role in vacuole isolation and heterocyclic organic anion detoxification [4,5]. The biosynthetic precursor of anthocyanin is phenylalanine, which is mainly synthesized through the metabolic pathway of flavonoids in the cytoplasm. The enzymes involved in the anthocyanin synthetic pathway loosely form a multi-enzyme complex—cinnamate-4-hydroxylase (C4H), flavanone-3b-hydroxylase (F3H), and flavonoid 3′-hydroxylase (F3′H)—that are used as scaffolds to assemble with soluble subunits on the endoplasmic reticulum. These enzymes are further regulated and modified by the ternary complex (MBW) composed of R2R3-MYB, bHLH, and WD40 proteins [6,7]. A special glutathione S-transferase (GST) is needed in the process of anthocyanin entering vacuoles [5].

As a representative of the multigene family, the GST enzyme family is quite complex (EC 2.5.1.18). It has an indispensable regulatory function in a large number of metabolic pathways [8,9]. For example, plant GSTs have good detoxification effects in exotic organisms, toxic lipid peroxides, and heavy metals [10,11,12,13]. The main function of GSTs is to catalyze glutathione (GSH), adding GSH to heterocyclic organic anions [14]. 

Plant GSTs play a crucial role in transporting anthocyanin into vacuoles, and they are important non-catalytic carrier proteins for the absorption of anthocyanin by vacuoles in plants [15]. According to the sequence correlation between members within the family, genome tissue specificity, and immunological characteristics, it can be divided into a total of 14 categories: Phi (F), Tau (U), Lambda (L), dehydroascorbate reductase (DHAR), Theta (T), Zeta (Z), elongation factor 1Bγ (EF1Bγ), tetrachloro hydroquinone dehalogenase (TCHQD), glutathionyl hydroquinone reductase (GHR), iota, mPGES-2, Ure2p, hemerythrin, and metaxin [16,17]. The GST protein contains two conserved domains. One is the conserved GSH-binding domain (G-site) at the N-terminus and the other is the substrate-binding domain (H-site) at the C-terminus. These two domains are close to each other through the three-dimensional (3D) structure to form catalytic sites with specific functions [18]. At present, many GST genes encoding anthocyanin transporters in different species have been reported: *BZ2* in maize [5], *TT19* in *Arabidopsis thaliana* [15,19], *VvGST1* and *VvGST4* in *Vitis vinifera* [20], *LcGST4* in *Litchi chinensis* Sonn. [21], and *FvRAP* in *Fragaria × ananassa* Duch. [22]. These studies suggest that GSTs are highly conserved in the anthocyanin synthetic pathway.

In this study, we identified 38 GSTs from the apple (*Malus domestica*) genome (HFTH1 Whole Genome v1.0) based on the sequence similarity with the GST family proteins of *Arabidopsis*. Subsequently, their structures, motifs, 3D models, and chromosomal distribution were further analyzed. Finally, we found cytosol-localized *MdGSTU12* is involved in the regulation of anthocyanin accumulation.

## 2. Materials and Methods

### 2.1. Plant Materials and Growth Conditions

Apple calli were obtained from the young embryos of the apple cultivar Orin (*Malus domestica*) and were cultivated on MS (Murashige and Skoog) medium supplemented with 1.5 mg/L 2,4-dichlorophenoxyacetic acid (2, 4-D) and 0.4 mg/L 6-benzylaminopurine (6-BA) for 15 days at 24 °C under dark conditions.

### 2.2. Identification and Characterization of Apple GST Family Genes

Sequences and annotations of the apple genome and the *MdGST* genes were obtained from HFTH1 genome V1.0.a1 (https://www.rosaceae.org/species/malus_x_domestica_HFTH1/genome_v1.0, accessed on 9 April 2019). Amino acid sequences of 64 GSTs families in *Arabidopsis* were downloaded from TAIR database (https://www.arabidopsis.org/, accessed on 30 September 2020) according to Gene IDs [23]. To ensure the reliability of the results, we used two methods (blastp and hmmsearch) to identify GST protein in apple. At first, the GST proteins in *Arabidopsis* were compared with the apple genome protein database (HFTH1 genome V1.0.a1; https://www.rosaceae.org/species/malus_x_domestica_HFTH1/genome_v1.0, accessed on 9 April 2019) based on BLASTp 2.9.0 (*E*-value < 1 × 10^−30^, Identity > 45%). Another way to identify all MdGST proteins, the GST-C domain from the Pfam database (Pfam number PF00043; http://pfam.xfam.org/, accessed on 30 October 2020) was used as the probe for Hidden Markov model (HMM) to search genome files downloaded from HFTH1 genome V1.0.a1 [24]. The domain of MdGST proteins were identified by WebCD-Search and SMART software [25]. Then, the amino acid sequences of MdGST family members were extracted and submitted to the line ProtParam tool (https://web.expasy.org/protparam/, accessed on 9 April 2019) calculating amino acids length, theoretical pI, and molecular weight.

### 2.3. Bioinformatic Analysis of MdGSTs

Sequence alignment and phylogenetic tree GST protein sequences of apple and *Arabidopsis* were aligned by ClustalW [26]. The reliability of the tree was assessed with 1000 bootstrap replicates, and the tree was drawn in MEGA-X software. To compare the evolutionary relationships and identify the subfamilies, the putative GSTs from apple, *Arabidopsis* and tomato were used to construct the molecular phylogenetic tree using MEGA-X with neighbor-joining (NJ) method [27]. iTOL online software (https://itol.embl.de/itol.cgi/, accessed on 8 April 2016) was used to decorate evolutionary trees [28].

According to the protein sequence of 38 MdGST, the three-dimensional MdGST protein structures were modeled using the online tool Phyre2 (http://www.sbg.bio.ic.ac.uk/phyre2/html/page.cgi?id=index, accessed on 7 March 2017). Gene Structure Display Serve 2.0 software (http://gsds.gao-lab.org/, accessed on 15 April 2015) was used to investigate the exon-intron organizations of *MdGST* genes based on the HFTH1 genome′s annotation file. Batch_SMART in TBTools (v0.6733) [29] was used to analysis domains and motif of MdGSTs, and the sequence was enriched by Weblogo3 (http://weblogo.threeplusone.com/create.cgi, accessed on 4 March 2019). Clustal Omega tool (https://www.ebi.ac.uk/Tools/msa/clustalo/, accessed on 1 July 2019) was used to complete multiple sequence alignment commands of MdGST protein [30]. Conserved protein domains of the MdGSTs were predicted using MEME (v.5.1.1, http://meme-suite.org/tools/meme, accessed on 25 August 2021) [31].

We got the apple HFTH1 genome annotation files, including *MdGST* gene location and structure. Chromosomal locations were drawn with MapGene2Chromosome V2 (http://mg2c.iask.in/mg2c_v2.0//, accessed on 19 November 2014). Interspecific collinearity analysis was based on apple and *Arabidopsis* genome sequences, and microsynteny analysis using TBtools based on GST ID and chromosome sequences in apple and *Arabidopsis* [29].

To analyze *cis*-elements in the *MdGST* promoters, we extracted 2000-bp long sequences upstream of the transcription start sites of the *MdGST* genes from the apple HFTH1 genomic sequence, and then used PlantCARE (http://bioinformatics.psb.ugent.be/webtools/plantcare/html/, accessed on 11 September 2000) to predict *cis*-acting element.

### 2.4. Construction of the Expression Vectors and Genetic Transformation

The sequence of *MdGSTU12* (*HF22792*) was inserted into a pCXSN-MYC vector to generate the *35S::MdGSTU12-OX*. The construct, *MdGSTU12-OX*, was transformed into *Agrobacterium* strain LBA4404, and the transgenic calli of apple was obtained on the basis of the method of Hu et al. [32]. The primers required for this experiment are listed in Table S1.

### 2.5. Viral Vector-Mediated Transient Expression in Apple Skins

Apple skin injection assays were performed as described previously [33]. *MdGSTU12-IL60* (IL60-1 + *MdGSTU12-IL60-2*) was overexpression vectors. *MdGSTU12-TRV* (*TRV1 + MdGSTU12-TRV2*) was suppression expression vectors. IL60 (IL60-1 + IL60-2) and TRV (TRV1 + TRV2) were empty vectors and used as references.

### 2.6. Quantitative Real-Time PCR Analysis

Total RNA was isolated using an RNAplant Extraction Kit (TIANGEN, Beijing, China). cDNA was synthesized from a reverse transcription kit (TaKaRa, Shiga, Japan). Quantitative primers of anthocyanin synthesis-related genes were the same as reported [34,35]. qRT-PCR was performed using the UltraSYBR mixture (High Rox) kit (ComWin Biotech Co., Ltd., Beijing, China) following the manufacturer’s instructions with 40 cycles for 15 s at 95 °C and 40 s at 60 °C on the real-time PCR system. The results were quantitatively analyzed by using the 2^−ΔΔCT^ method. The *18S* gene was used as an internal control. 

### 2.7. Subcellular Localization of MdGSTU12

The full-length coding sequences of *MdGSTU12* was fused to the GFP protein to contruct the fusion expression vector *35S::MdGSTU12-GFP*, and the resulting plasmid was transformed into *Agrobacterium* strain LBA3101. Inject the constructed vector into tobacco (*Nicotiana benthamiana*) epidermal cells and cultured in the dark for three days. Fluorescence images were obtained at 488 nm with a high-resolution laser confocal microscope (LSM880, Zeiss, Meta, Jena, Germany).

### 2.8. Determination of Anthocyanin Extraction and Measurement 

Total anthocyanin in apple calli were extracted via the methanol-HCl method [35]. The plant materials were incubated in anthocyanin extraction solution with 95% absolute enthanol and 1.5 M HCl at room temperature for 24 h. The absorbance values of extracted anthocyanin were determined by ultraviolet spectrophotometer (SOPTOP, Shanghai, China) at 530, 620, and 650 nm. Calculation of anthocyanin content was conducted by previously described methods [36].

### 2.9. Statistical Analysis

All experiments were performed in triplicates. Error bars show standard deviation of three replicates. Significant difference was detected by *t*-test using GraphPad Prism 6.02 software (*, *p* < 0.05; **, *p* < 0.01). 

## 3. Results

### 3.1. Identification and Bioinformatic Analysis of MdGSTs 

To determine the characteristic function and special properties of the GST family, and more accurately find each member of the GST family, we used the *Arabidopsis* GST protein sequences. The GST family members in the apple HFTH1 genome were strictly screened through the blastp and hidden Markov model (HMM) searches [24], which finally accurately identified 38 GST family members. The *MdGST* family was classified and named according to the evolutionary homology of the GST family between *Arabidopsis* and apple. According to the gene annotation information, the length of the *MdGST* genes varied from 522 bp to 4983 bp, encoding 173 to 1660 amino acids. The predicted molecular weights were between 19.99 KDa and 186.83 KDa, and the predicted theoretical isoelectric points ranged from 5.17 to 9.68 (Table S2).

**Figure 1:**
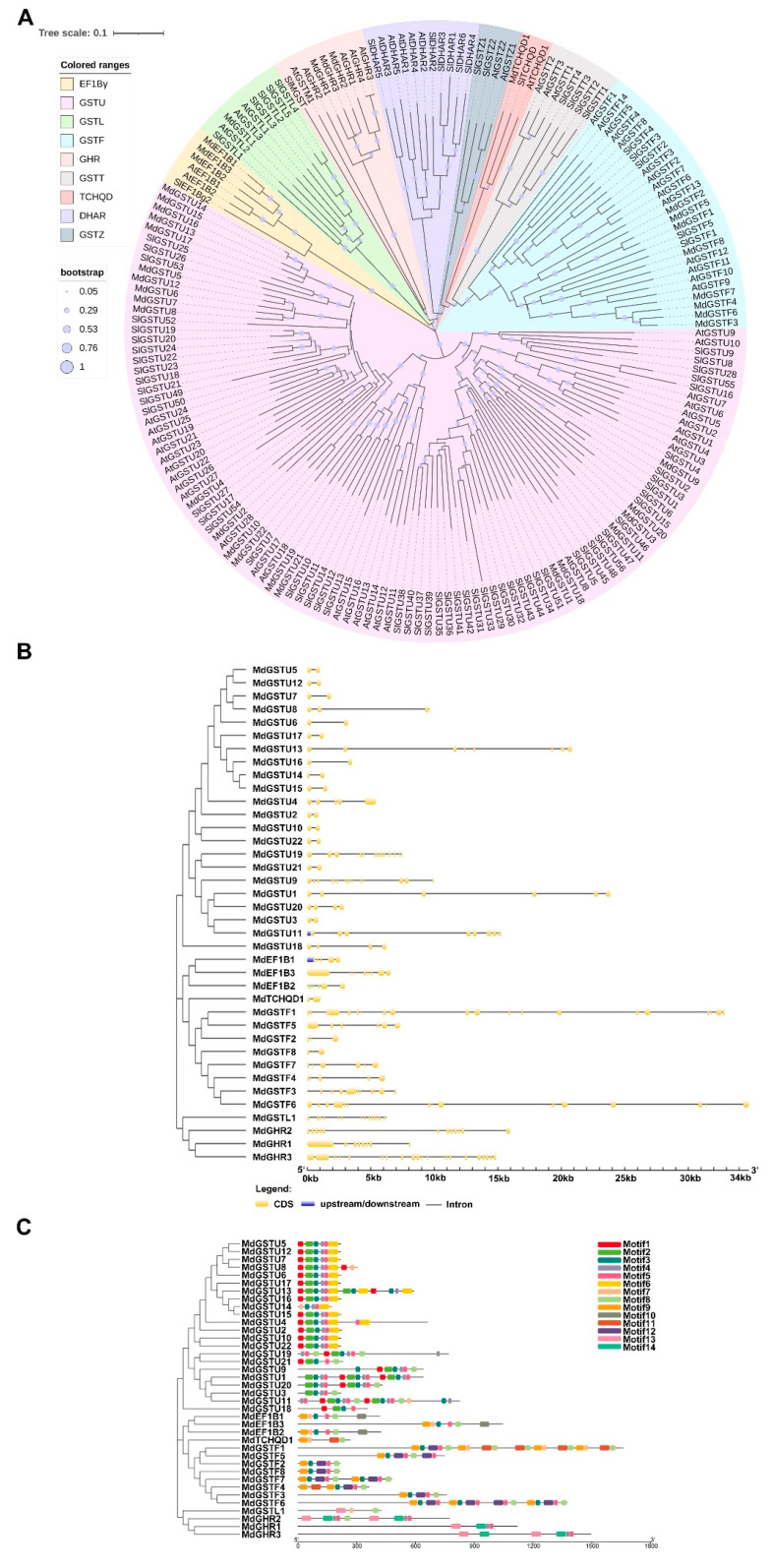
Phylogenetic analysis, gene structure, and motif analysis of the MdGSTs. (**A**) The phylogenetic analysis of GST family genes of *Arabidopsis*, apple, and tomato. The tree was constructed via the maximum likelihood method with 1000 bootstrap replications. A neighbor-joining tree was generated by MEGA X software, and the tree was annotated using Interactive Tree Of Life (iTOL). The solid line represents the real branch length, and the dotted lines were added later for better visualization. (**B**) Exon-intron structure analysis of *MdGST* genes. The exons and introns are represented by yellow boxes and black lines, respectively. (**C**) Conserved domains of the MdGSTs, represented by colored blocks.

To explore the phylogenetic relationship of MdGST proteins, we used MEGA software to construct a phylogenetic tree of 64 *A. thaliana*, 38 apple, and 81 tomato GST protein sequences with the help of maximum likelihood method (bootstrap = 1000) (Figure 1A). The phylogenetic tree results showed that apple GST proteins had high homology with those of *A. thaliana* and tomato. The apple GST proteins were divided into nine classes (U, F, L, Z, T, GHR, EF1Bγ, TCHQD, and DHAR) based on the previous reports (Figure S1). Amidst these, the Tau subfamily was the largest group and accounted for more than half of the total number of GSTs in the studied species. Amongst the 38 MdGSTs in apple, 22 proteins (MdGSTU1-22) were classified into Tau, 8 proteins (MdGSTF1-8) were classified into Phi, and 3 proteins (MdEF1B1-3, MdGHR1-3) were classified into EF1Bγ and GHR respectively. Only one protein (MdGSTL1, MdTCHQD1) was classified into Lambda and TCHQD respectively.

To analyze the evolutionary relationship of MdGST proteins, the neighbor-joining phylogenetic tree was repeatedly constructed using the maximum likelihood method, so that MdGST was clustered and distributed. The introns and exons of the 38 apple *GST* genes were analyzed later. The results showed that apple *GST* genes contained two or more introns. In the Tau subfamily, most apple *GST* genes contained two exons and one intron except for some genes. There were many exons in the EF1Bγ, Phi, Lambda, and GHR subfamilies, of which *MdGSTF1* contained the largest number (24) of exons (Figure 1B). To further understand the function of apple GST proteins, we identified 14 conserved motifs in MdGSTs using the MEME website, and found similar motifs in the same subfamily, indicating that they may have extensive similar functions (Figure 1C). These results were compared and analyzed by SMART in NCBI, and the predicted motifs were annotated. Motifs 2, 3, and 4 were annotated as the GST-N domain, and motifs 5 and 6 were annotated as the GST-C domain (Figure S1A).

To compare the structural characteristics of the two domains (GST-C and GST-N) in MdGST proteins, Phyre was used to precisely construct the tertiary structures of MdGSTs (Figure S1B). The results showed that the structures of MdGST proteins in the same subfamily and those sharing close genetic relationships were highly similar. It was well known that the structures and functions of proteins were closely related. This also indicated that the MdGST proteins from the same subfamily retained some similar functions in the evolution of apple.

### 3.2. Chromosomal Locations and Collinearity Analysis

**Figure 2:**
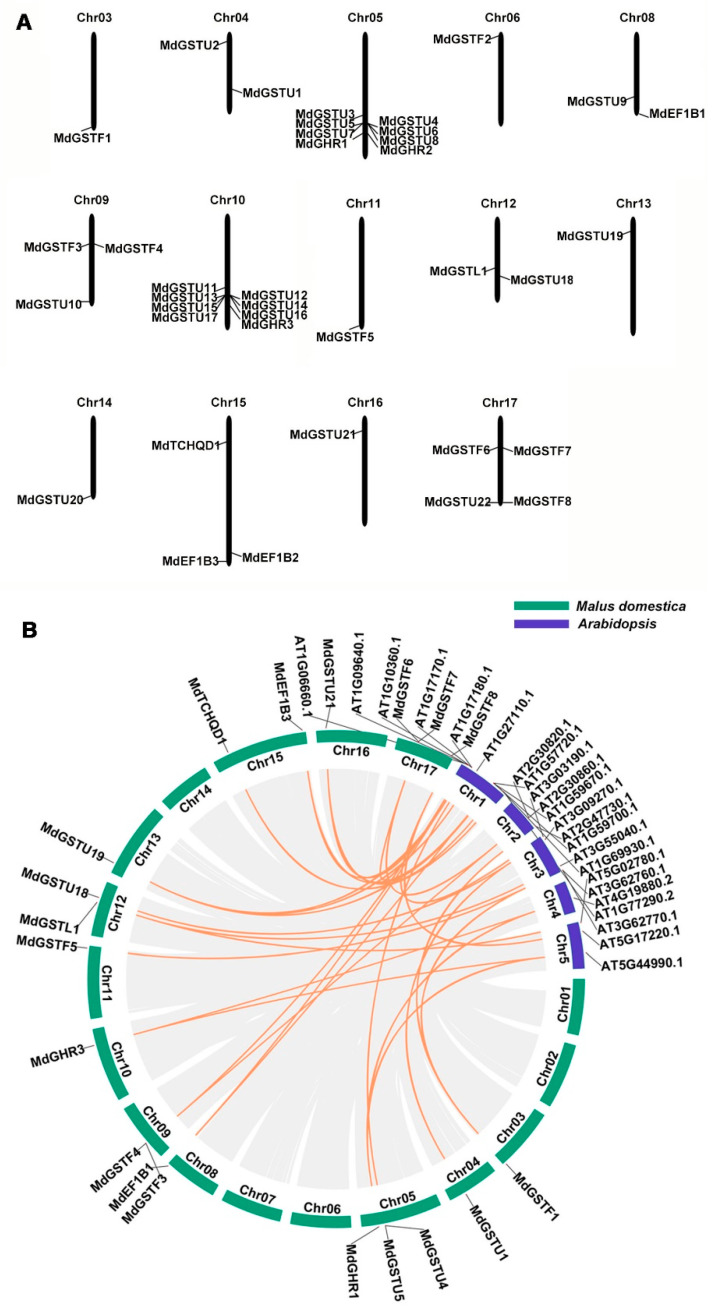
Chromosomal locations and collinearity analysis of the *MdGSTs*. (**A**) Chromosomal locations of the 38 *MdGSTs* in apple, which are based on the physical positions (Mb) of genes from the apple HFTH1 genome. *MdGSTs* were located on 14 of the 17 apple chromosomes. The chromosome number is shown at the top of each chromosome. (**B**) Collinearity analysis of GSTs among apple and *Arabidopsis* chromosomes.

By observing the distribution of *MdGSTs* in the apple HFTH1 genome, the location of each *MdGST* on apple chromosomes was determined. Chromosomal localization analysis showed that the 38 *MdGST* genes were distributed on 14 chromosomes. Amongst them, *MdGST* genes were mainly distributed on Chr05 and Chr10, and each of them had 8 *MdGST* genes. There were three *MdGST* genes on Chr09 and Chr15 respectively; and two *MdGST* genes on Chr04, Chr08, and Chr12, respectively. There was only one *MdGST* gene on Chr03, Chr06, Chr11, Chr13, Chr14, and Chr16, respectively, while there was no *MdGST* gene distributed on Chr01, Chr02, or Chr07 (Figure 2A). In addition, we wanted to determine whether *MdGST* located on Chr05 and Chr10 contained apple molecular markers (SNPs or QTLs), but we did not find the HFTH1 database that could directly search for SNPs, and we found the GDDH13 database that could search for SNPs in GDR (https://www.rosaceae.org/search/markers, accessed on 8 September 2020). The 16 genes (*MdGSTU3*-*MdGSTU8*, *MdGSTU11*-*MdGSTU17*, *MdGHR1*-*MdGHR3*) located on Chr05 and Chr10, and some genes with the highest homology with each other using blastp method in the GDDH13 database. Then, according to the chromosome positions of these genes, two genes were found to contain SNP. There were four SNPs in *MD05G1209700* (*HF11214*) and six SNPs in *MD05G1252400* (*HF11551*). The details are shown in Table S3.

To reveal the expansion mechanism of the GST family, all intergenomic duplication data files of apple and *Arabidopsis* were filtered by TBtools [29]. Microsynteny between species can be used to identify the location of orthologous genes. In total, we identified 32 orthologous gene pairs between apple and *Arabidopsis* (Table S4). This indicates that the numerous apple and *Arabidopsis* counterparts appeared to be derived from a common ancestor (Figure 2B).

### 3.3. Analysis of Cis-Regulatory Elements of MdGSTs and Expression Profiles of MdGST Genes in Four Developmental Stages of the ‘Gala’ Strain

**Figure 3:**
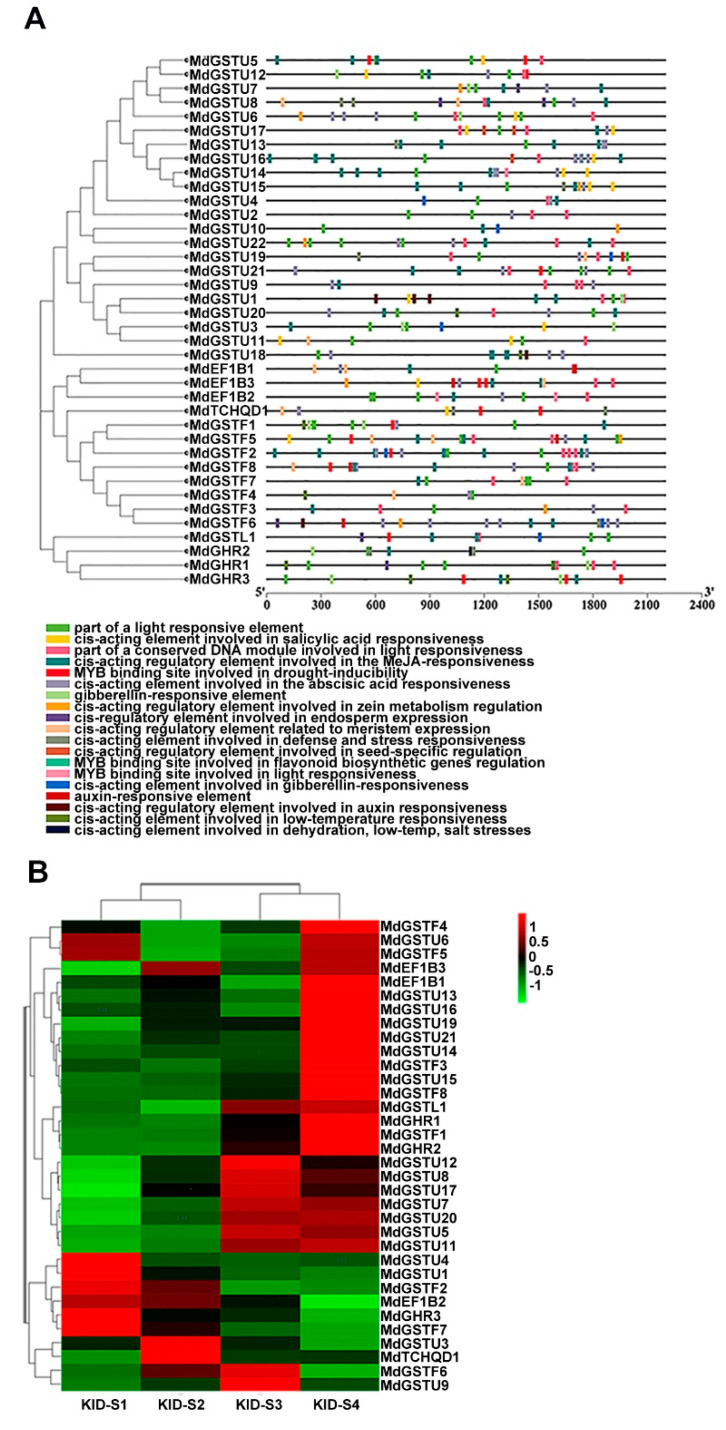
The main *cis*-acting elements in *MdGST* promoters and expression patterns of *MdGST* genes during fruit development. (**A**) Promoter analysis was performed on 2000-bp sequences upstream of the transcription start sites. (**B**) KID-S1 (85 days after flowering), KID-S2 (107 days after flowering), KID-S3 (128 days after flowering), and KID-S4 (145 days after flowering) represent the four main stages of anthocyanin accumulation during fruit developmental stages, KID represents the ‘Gala’ strain. The color in the Figure 3B indicates the expression level, which is the normalized value of pHeatmap in R language of Log 2 FC. The deeper the red is, the higher the expression is. The deeper the green is, the lower the expression is.

The cis-acting elements of the *MdGST* promoter were analyzed by Plant CARE. This analysis included hormone-related responsive elements such as gibberellic acid (GA), saliycilic acid (SA), jasmonic acid (JA), and auxin; stress-related responsive elements such as low temperature and drought; and responsive elements involving genes related to flavonoid biosynthesis. It also indicated that *MdGSTs* likely to play a role in response to these hormones, stress conditions, and flavonoids such as anthocyanins (Figure 3A). Many studies have shown that GST is a crucial transporter involved in anthocyanin accumulation [21,22,37]. To explore the close relationship between *MdGSTs* and anthocyanin metabolic pathways, we used RNA-seq data from previous studies. The four different developmental stages S1, S2, S3, and S4 (covered the period from small fruit to harvest including 85, 107, 128, 145 days after blooming) of the ‘Gala’ strain apple (KID) were analyzed. Previous studies have shown that the stage from S2 to S3 is the key period of anthocyanin accumulation [38]. 

Hence, we analyzed the expression of the *MdGST* family in the four stages (S1, S2, S3, and S4) of the ‘Gala’ strain (Figure 3B). Amongst the genes with high expression levels, nine *MdGST* genes (*MdGSTU12*, *MdGSTU8*, *MdGSTU17*, *MdGSTU7*, *MdGSTU20*, *MdGSTU5*, *MdGSTU11*, *MdGSTF6*, *MdGSTU9*) were upregulated from S2 to S3, suggesting that these genes are related to anthocyanin accumulation in apple. According to the color change, the more obvious changes in expression was *MdGSTU12*. Finally, we selected a gene *MdGSTU12* with more obvious upregulation from S2 to S3 for further study.

### 3.4. MdGSTU12 Expression Positively Correlates with Anthocyanin Content and Anthocyanin Synthesis Related Genes

**Figure 4:**
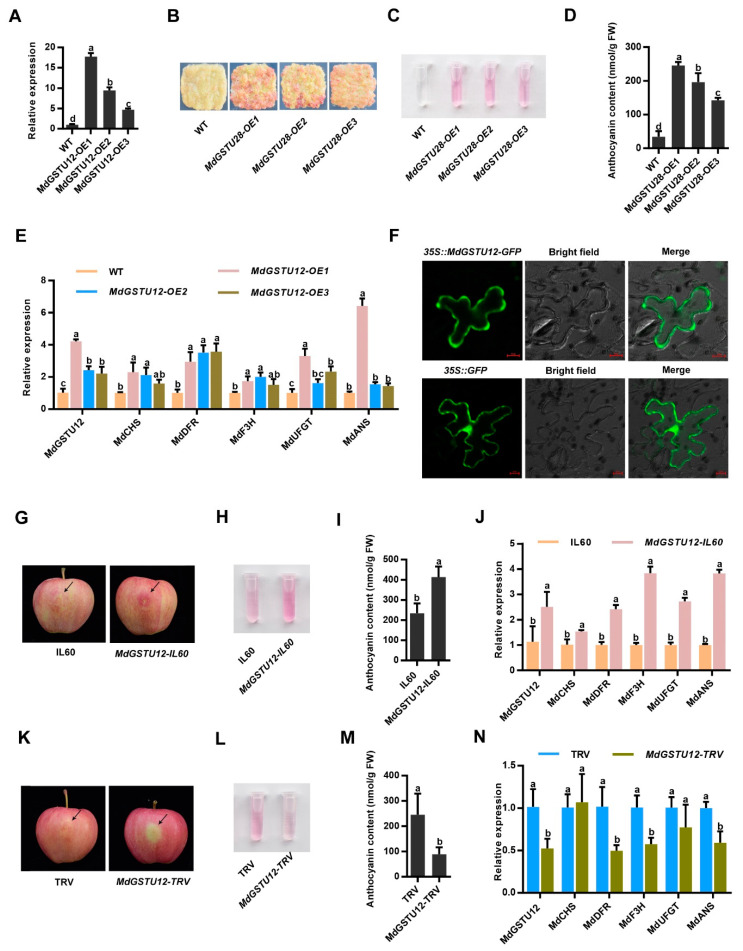
*MdGSTU12* is involved in the regulation of anthocyanin accumulation and subcellular localization of MdGSTU12 protein. (**A**) The expression of *MdGSTU12* in the wild-type (WT) and *MdGSTU12* transgenic apple calli by RT-qPCR assay. (**B**,**C**) Calli coloration of two-week-old WT, *MdGSTU12-OE1*, *MdGSTU12-OE2*, and *MdGSTU12-OE3* transgenic calli. (**D**) Anthocyanin contents in the apple calli are shown in (**B**). (**E**) Expression analysis of *MdGSTU12* and the anthocyanin biosynthesis-related genes *MdCHS*, *MdDFR*, *MdF3H*, *MdUFGT*, and *MdANS* by RT-qPCR assays in (**B**). (**F**) Subcellular localization of *MdGSTU12* protein. *35S::GFP* served as a control, scale bar = 10 µm, BF represents a bright field. (**G**) Coloration of apple fruit peels injected with plasmid mixtures (IL60: IL60-1 + IL60-2; *MdGSTU12-IL60*: *IL60-1 + MdGSTU12-IL60-2*). An empty IL60 vector was used as control. (**H**,**J**) Anthocyanin content (**H**,**I**) and relative expression levels of the anthocyanin biosynthesis-related genes (**J**) around the injection sites of the fruit peels shown in (**G**). (**K**) Coloration of apple fruit peels injected with a mixed solution of Agrobacterium cells (TRV: TRV1 + TRV2; *MdGSTU12-TRV*: *TRV1 + MdGSTU12-TRV2*). An empty TRV vector was used as a control. (**L**–**N**) Anthocyanin contents (**L**,**M**) and transcript levels of anthocyanin biosynthesis-related genes (**N**) around the injection sites of the fruit peels shown in (**K**). The *18s* gene acted as the internal control. In A, D, E, I, J, M, N, the error bars indicate the standard deviation (SD) of three independent experiments, each of which included three technical replicates. Different lowercase letters indicate a significant difference at *p* < 0.05.

To confirm that *MdGSTU12* was involved in the regulation of anthocyanin content in apple, Orin apple calli (WT) and *MdGSTU12* transgenic calli (*MdGSTU12*-*OX*) were used for calli coloring experiments. Figure 4A confirmed that we obtained the overexpression line of *MdGSTU12*. According to the results, it can be seen that overexpression of *MdGSTU12* significantly correlates with the accumulation of anthocyanin (Figure 4B,C), and anthocyanin content values shown in Figure 4D. 

In addition, we further detected the expression level of anthocyanin biosynthesis-related genes *MdCHS*, *MdDFR*, *MdF3H*, *MdUFGT*, and *MdANS* according to the anthocyanin biosynthesis pathway (Figure S2). Expression analysis showed that the expression levels of the anthocyanin biosynthesis-related genes were upregulated in *MdGSTU12* transgenic calli (Figure 4E). It can be seen from the anthocyanin biosynthesis pathway that when *MdGSTU12* is overexpressed, more anthocyanin will be transported into the vacuole, thereby reducing the amount of anthocyanin that has not been transported to the vacuole. In order to achieve the normal transportation of anthocyanin, negative feedback regulation will be carried out to stimulate the expression of upstream synthesis-related genes and generate more anthocyanin, resulting in an increase in anthocyanin that is stable in the vacuole and ultimately promotes the accumulation of color. Therefore, these results confirmed that *MdGSTU12* was involved in the regulation of anthocyanin accumulation in apple.

According to previous studies, GSTs exist in the cytoplasm as an anthocyanin transporter to promote anthocyanin accumulation [39]. We further determined the intracellular localization of *MdGSTU12* using fused GFP as an indicator. *35S::GFP* was used as a control, which was distributed in the nucleus and cytoplasm. The localization of *MdGSTU12-GFP* under a confocal microscope showed that the edge of *MdGSTU12-GFP* was discontinuous, which conforms to the characteristics of cytoplasmic localization (Figure 4F).

To further explore the role of *MdGSTU12* in apple peel coloration, viral vector-mediated transient injection assays were carried out in apple skins. As a result, overexpression of *MdGSTU12* promoted anthocyanin biosynthesis in apple skins around the infiltration sites (Figure 4G–I), and suppression of *MdGSTU12* expression inhibited the biosynthesis compared with that of the controls (Figure 4K–M). Moreover, the trends of the relative expression levels of the anthocyanin biosynthesis-related genes were basically similar to the trends of anthocyanin accumulation in the injected fruit peels (Figure 4J,N). These results indicated that *MdGSTU12* promoted anthocyanin accumulation by regulating the expression of anthocyanin synthesis-related genes.

## 4. Discussion 

Anthocyanins are synthesized in the cytoplasm through flavonoid metabolic pathways and finally transported to vacuoles for storage [39]. The intracellular transport mechanism of anthocyanin has been revealed in previous studies. Anthocyanins entering vacuoles from the cytoplasm requires GST mediation, membrane transport, or vesicles trafficking [40]. GSTs are multifunctional enzymes involved in secondary metabolites. The involvement of GSTs in anthocyanin accumulation has been testified in *Arabidopsis* [41], peach [37], litchi [21], cyclamen [42], and strawberry [43]. In the current research, we manifested that the *MdGSTU12* gene from apple encoded a GST. Interestingly, we found that *MdGSTU12* expression positively correlates with anthocyanin content and anthocyanin synthesis related genes in this study; this offers data for a new survey of the molecular mechanisms of anthocyanin accumulation in apple.

GST is a supergene family in higher plants, which is separated into U, F, L, Z, T, GHR, EF1Bγ, TCHQD, and DHAR subclasses. Until now, numerous GSTs are found in plants: 64 GSTs in *Arabidopsis*, 139 GSTs in litchi, and 82 GSTs in radish [21,23,44]. The present research suggests that 38 GSTs were found in apple HFTH1 genome (Table S1). *MdGSTU12* belonged to the Tau subclass, which is the same subclass known for anthocyanin-related GSTs in maize [5]. This confirms that GSTs are highly conserved in evolution. 

Owing to the significance of GSTs in anthocyanin accumulation, numerous studies investigated the factors affecting GST expression. Several internal elements affecting GST expression have been identified. In this study, some hormone-responsive, stress-responsive, and responsive elements involving genes related to flavonoid biosynthesis were predicted in the promoter of *MdGSTs* (Figure 3A), implying that the expression of *MdGSTs* is possibly regulated by an internal element.

To accurately explore the genes affecting anthocyanin accumulation in apple, the expression profiles of *MdGSTs* during fruit ripening of apple were analyzed (Figure 3B). We revealed that the expression level of *MdGSTU12* increased significantly during the major period of apple fruit coloring. In the present study, we showed that *MdGSTU12* promoted anthocyanin biosynthesis in transgenic calli and apple fruits (Figure 4A–E,G–N).

It is generally recognized that the function of proteins is closely related to the subcellular localization [45]. We confirmed that *MdGSTU12* was located in the cytoplasm (Figure 4F), which was consistent with the important process of GSTs participating in anthocyanin accumulation as transporters in the cytoplasm. 

The accumulation of anthocyanin in apple fruit will affect the coloring of fruit. As an important appearance quality, fruit color has high commercial value. Producing brightly colored apple fruit is also an important goal of apple breeding. Although anthocyanin synthesis pathway has been largely resolved, study of the GSTs transport anthocyanin pathway remains to be strengthened. Therefore, it is of far-reaching significance to find the factors affecting anthocyanin transport for the analysis of apple coloring regulation network. In this study, we confirmed that *MdGSTU12* can participate in the anthocyanin synthesis pathway and promote the accumulation of anthocyanin, which lays the foundation for further study of the specific regulatory network of GSTs involved in anthocyanin accumulation.

## 5. Conclusions

In summary, we identified 38 GSTs from the apple HFTH1 genome. Detailed bioinformatic analyses were carried out on phylogenetic relationships, gene structures, motifs, 3D models, *cis*-acting elements, chromosomal locations, collinearity, and expression patterns of *MdGST* genes. We also used the traditional method to determine the significance of *MdGSTU12* in apple anthocyanin accumulation. These results suggest that *MdGSTU12* might play an important role in the regulation of anthocyanin in apple.

## Data Availability

Not applicable.

## Supplementary Materials

The following are available online at https://www.mdpi.com/article/10.3390/genes12111733/s1, Figure S1: Protein sequences of the conserved domains and three-dimensional models of the MdGSTs; Figure S2. Schematic representation of the anthocyanin biosynthesis pathway; Table S1. Primers used in this study; Table S2. The information of GST family genes in apple; Table S3. Primers used in this study; Table S4. Intergenomic duplicated MdGST genepairs; Appendix S1. Sequences used for phylogenetic trees and sequence alignment.
