# Supplementary material for: Genome-Wide Analysis of the Glutathione S-Transferase (GST) Genes and Functional Identification of MdGSTU12 Reveals the Involvement in the Regulation of Anthocyanin Accumulation in Apple
Source: Genes (Basel). 2021 Oct 29;12(11):1733. doi: 10.3390/genes12111733 (PMC8619396; doi:10.3390/genes12111733)
Supplement: Supplementary file 1 [file genes-12-01733-s001.zip › genes-1432877-supplementary.pdf]

Genes Supporting Information Figures S1–S2, Tables S1–S4 and Appendix S1

The Title:

Genome-wide analysis of the glutathione S-transferase (GST) genes and functional identification of *MdGSTU12* reveals the involvement in the regulation of anthocyanin accumulation in apple

The full names of all the authors:

Yu-Wen Zhao, Chu-Kun Wang, Xiao-Yu Huang, Wen-Li Lu*, Cui-Hui Sun*, Da-Gang Hu*

The names and address of the institution:

National Key Laboratory of Crop Biology; Shandong Collaborative Innovation Center of Fruit & Vegetable Quality and Efficient Production; College of Horticulture Science and Engineering, Shandong Agricultural University, Tai'an, Shandong 271018, China

Corresponding authors:

Da-Gang Hu (fap_296566@163.com); Cui-Hui Sun (suncuihui@163.com); Wen-Li Lu (365303776@qq.com)

Tel: +86-538-824-6151

Fax number: +86-538-824-6151

Address: College of Horticulture Science and Engineering, Shandong Agricultural University, Tai'an 271018, Shandong, China

Figure S1. Protein sequences of the conserved domains and three dimensional models of the MdGSTs. A. Protein sequences of the conserved domains in Figure 1C. B. Three-dimensional models of the MdGST proteins.

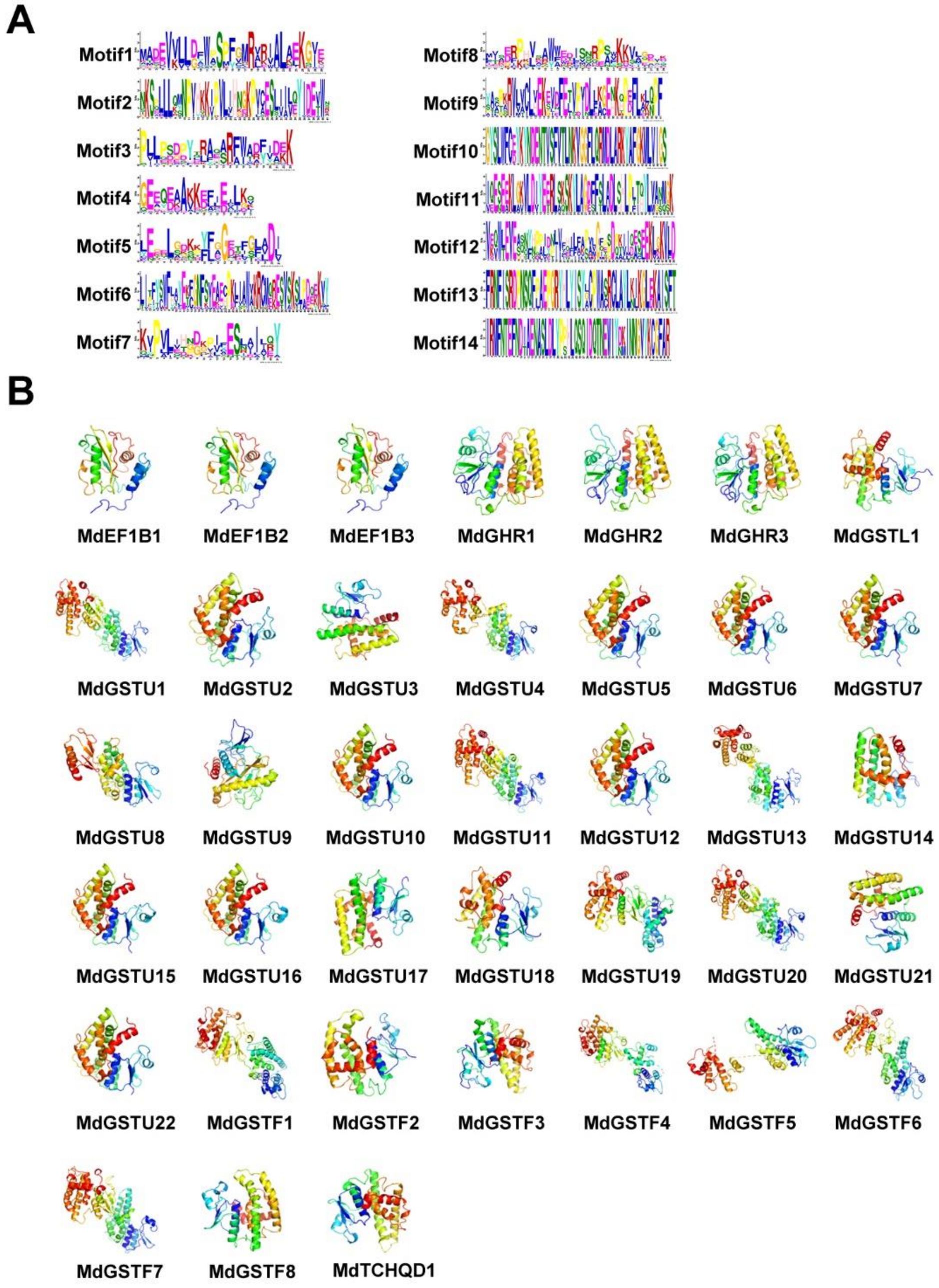

Figure S2. Schematic representation of the anthocyanin biosynthesis pathway. CHS, chalcone synthase; CHI, chalcone isomerase; F3H, flavanone 3-hydroxylase; DFR, dihydroflavonol reductase; ANS, anthocyanidin synthase; UFGT, 3-O glucosyltransferase.

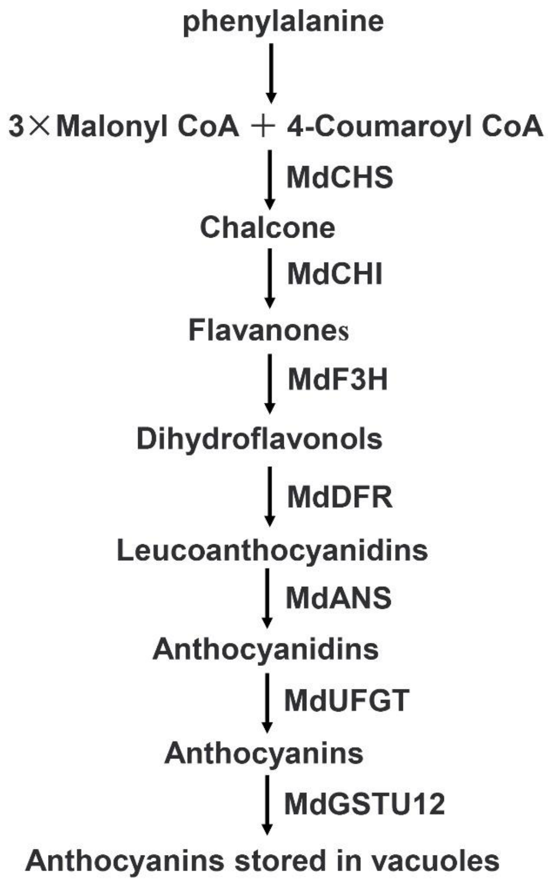

Table S1. Primers used in this study.

Gene name	Prime sequence	Annotation
MdCHS-F	CGCCTCCACTACAACCTCC	
MdCHS-R	GGCAAGTGCTGTCGGATT	
MdDFR-F	GGTAAATGTAAAACAATAGAGAGG	
MdDFR-R	GGAAGTGGTTTTGTCGCCTG	
MdF3H-F	GCCGATCACCTACACCGAG	
MdF3H-R	GTACAAGAAGTGGGAAGGC	
MdCHI-F	GGGATAACCTCGCGGCCAAA	
MdCHI-R	GCATCCATGCCGGAAGCTACAA	Primers for
MdANS-F	GGAGAAGATCATCCTTAAGCCA	quantative
MdANS-R	CTAAGATATATCATACCAACTATGCC	RT-PCR in
MdUFGT-F	GGAAGTGGTTTTGTCGCCTG	apple
MdUFGT-R	CATTATTATTGAGCAACGAACAGC	
qRT-MdGSTU12-F	GTTGGAAAGTGCCCTTGCTG	
qRT-MdGSTU12-F	CCTCGACGATTCTAGCCCAC	
18S-F	ACACGGGGAGGTAGTGACAA	
18S-R	CCTCCAATGGATCCTCGTTA	
MdGSTU12-F	ATGTCTGGAGAATCAGTGAAATTGCTT	Primers for
MdGSTU12-R	ACTTGATGCTGCTGTCAAAATCTT	MdGSTU12
MdGSTU12(pCXSN)-F	AATGTCTGGAGAATCAGTGAAATTGCTT	Primers for
MdGSTU12(pCXSN)-R	ACTTGATGCTGCTGTCAAAATCTT	transgene
pIR-MdGSTU12-F	GTCGACATGTCTGGAGAATCAGTGAAATTGCTT	Primers for subcellular
pIR-MdGSTU12-R	TCTAGAACTTGATGCTGCTGTCAAAATCTT	localization

Table S2. The information of GST family genes in apple.

	Gene	Gene ID	Location	Homologous	Amino acids length	Theoretical pI	Molecular weight (average)
	MdEF1B1	HF34534	Chr08: 30862280..30864889	AtEF1B2	419	6.58	47568.86
EF1Bγ	MdEF1B2	HF17304	Chr15: 51503535..51506505	AtEF1B1	424	6.98	48314.59
	MdEF1B3	HF17033	Chr15: 54823425..54829995	AtEF1B2	1046	8.78	118859.14
	MdGHR1	HF11550	Chr05: 38195701..38203818	AtGHR3	1120	8.71	127244.07
GHR	MdGHR2	HF11551	Chr05: 38214218..38230210	AtGHR2	774	5.39	86658.18
	MdGHR3	HF23084	Chr10: 34645358..34660230	AtGHR3	1496	8.08	168491.33

GSTF	MdGSTF1	HF02809	Chr03: 35861571..35894420	AtGSTF8	1660	6.56	186834.95
	MdGSTF2	HF10119	Chr06: 1553319..1555769	AtGSTF13	217	6.13	24284.89
	MdGSTF3	HF34035	Chr09: 11285864..11292861	AtGSTF11	759	5.98	85038.62
	MdGSTF4	HF34036	Chr09: 11292908..11299034	AtGSTF10	364	5.77	42246.51
	MdGSTF5	HF28390	Chr11: 41090504..41097823	AtGSTF13	748	5.76	83090.45
	MdGSTF6	HF29096	Chr17: 11943180..11977948	AtGSTF9	1375	6.45	157208.43
	MdGSTF7	HF29092	Chr17: 12017672..12023249	AtGSTF9	479	6.43	55462.92
	MdGSTF8	HF32109	Chr17: 32774782..32776133	AtGSTF12	215	5.67	24490.29
Lambda	MdGSTL1	HF04750	Chr12: 19273485..19279723	AtGSTL1	425	7.11	47953.59
GSTU	MdGSTU1	HF27822	Chr04: 21483651..21507519	AtGSTU8	640	5.27	73913.28
	MdGSTU2	HF00984	Chr04: 3554945..3555810	AtGSTU21	225	6.95	26243.57
	MdGSTU3	HF10959	Chr05: 31390805..31391664	AtGSTU22	221	5.47	25224.02
	MdGSTU4	HF11214	Chr05: 34330799..34336202	AtGSTU23	663	5.29	76222.02
	MdGSTU5	HF11218	Chr05: 34355206..34356206	AtGSTU19	219	5.38	25357.42
	MdGSTU6	HF11222	Chr05: 34387386..34390609	AtGSTU28	220	6.02	25614.82
	MdGSTU7	HF11223	Chr05: 34413627..34415510	AtGSTU25	219	5.89	25484.56
	MdGSTU8	HF11226	Chr05: 34433199..34442847	AtGSTU21	306	5.17	35527.98
	MdGSTU9	HF35045	Chr08: 24301568..24311504	AtGSTU1	641	5.8	72669.02
	MdGSTU10	HF36976	Chr09: 32991881..32992885	AtGSTU27	220	5.4	25031.99
	MdGSTU11	HF29919	Chr10: 27809939..27825187	AtGSTU8	827	8.51	94899.23
	MdGSTU12	HF22792	Chr10: 30650838..30651918	AtGSTU24	219	5.41	25510.73
	MdGSTU13	HF22793	Chr10: 30652608..30673444	AtGSTU25	592	6.23	68669.26
	MdGSTU14	HF22794	Chr10: 30695582..30696947	AtGSTU26	173	6.11	19991.22
	MdGSTU15	HF22797	Chr10: 30723612..30725202	AtGSTU21	221	6.15	25623.78
	MdGSTU16	HF22798	Chr10: 30750446..30753987	AtGSTU21	221	6.25	25854.04
	MdGSTU17	HF22799	Chr10: 30765908..30767210	AtGSTU19	219	6.03	25392.42
	MdGSTU18	HF06446	Chr12: 22326639..22332868	AtGSTU8	356	8.93	40583.88
	MdGSTU19	HF42508	Chr13: 5466824..5474320	AtGSTU17	769	5.59	86228.25
	MdGSTU20	HF24574	Chr14: 30174194..30177073	AtGSTU8	432	5.37	49561.35
	MdGSTU21	HF07438	Chr16: 5638898..5640039	AtGSTU17	231	6.85	25314.32
	MdGSTU22	HF32118	Chr17: 32639204..32640270	AtGSTU27	220	5.41	24970.1
TCHQD	MdTCHQD1	HF12597	Chr15: 9916687..9917733	AtTCHQD1	267	9.68	31444.51

Table S3. Primers used in this study.

HFTH1	GDDH13	Genen Location	Type	Map	SNP Location
HF11214	MD05G1209700	Chr05:34122009..34124732	SNP	Apple-MM-F1	Chr05:34123019..34123019
			SNP	Apple-MM-F1	Chr05:34124731..34124731
			SNP	Apple-M27xM116-2016	Chr05:34123019..34123019
			SNP	Apple-M27xM116-2016	Chr05:34124731..34124731
HF11551	MD05G1252400	Chr05:38385538..38388983	SNP	Apple-NC-F1	Chr05:38388831..38388831
			SNP	Apple-NC-F1	Chr05:38388832..38388832
			SNP	Apple-ZF-F1	Chr05:38388831..38388831
			SNP	Apple-ZF-F1	Chr05:38388832..38388832
			SNP	Apple-BB-F1	Chr05:38388831..38388831
			SNP	Apple-BB-F1	Chr05:38388832..38388832

Table S4. Intergenomic duplicated MdGST genepairs.

Apple Gene Name	Apple Gene ID	Arabidopsis Gene ID
MdGSTF1	HF02809	AT2G47730.1
MdGSTF1	HF02809	AT3G62770.1
MdGSTU1	HF27822	AT3G09270.1
MdGSTU4	HF11214	AT1G17170.1
MdGSTU5	HF11218	AT1G17180.1
MdGHR1	HF11550	AT4G19880.2
MdGHR1	HF11550	AT5G44990.1
MdEF1B1	HF34534	AT1G57720.1
MdEF1B1	HF34534	AT1G09640.1
MdGSTF3	HF34035	AT1G06660.1
MdGSTF3	HF34035	AT2G30820.1
MdGSTF4	HF34036	AT2G30860.1
MdGSTU19	HF42508	AT1G27110.1
MdGSTU19	HF42508	AT1G10360.1
MdGSTU19	HF42508	AT1G59700.1
MdTCHQD1	HF12597	AT1G77290.2
MdEF1B3	HF17033	AT1G57720.1
MdEF1B3	HF17033	AT1G09640.1
MdGSTU21	HF07438	AT1G69930.1
MdGSTU21	HF07438	AT1G10360.1
MdGSTU21	HF07438	AT1G59670.1
MdGSTF6	HF29096	AT1G06660.1
MdGHR3	HF23084	AT4G19880.2
MdGHR3	HF23084	AT5G44990.1
MdGSTF5	HF28390	AT3G62760.1
MdGSTL1	HF04750	AT3G55040.1
MdGSTU18	HF06446	AT3G09270.1
MdGSTL1	HF04750	AT5G02780.1
MdGSTF6	HF29096	AT2G30820.1
MdGSTF7	HF29092	AT2G30860.1
MdGSTF8	HF32109	AT3G03190.1
MdGSTF8	HF32109	AT5G17220.1

Appendix S1. Sequences used for phylogenetic trees and sequence alignment.

>HF02809 MdGSTF1

MWGMTKALSYPRISPRFQFDKLRFLNNQDWYDMFQPNFKVHLARCSTIIK
LYMRDILCSNIVMAIQVVQPVKKMYFGFEMNAVFVGGVVEQNRLHLYND
TLLPIFASKTYLLLPNPEKAESRTLSSSGSRSSKRTPQNPQKSHHPDPPPPMA
TLSAFSPPSWPDPNSAADFLTDPTDPTDENPTSVVDSSDDHHDPQSPPNPN
HNLHNNHPFSSSPPEPAPPAPRSPPSLFHVCFNQDHSCFAVGTDHGFRIYN
CDPFRELFRRDFDNGGGIGVVEMLFRCNILAIVGGGPDPQYPTNKVMIWD
DHQERCIGELSFRSFVRSVRLRRDRIVVVLEQKIFVYNFADLKLLHQIETIAN
PKGLCAVSQVARSLVLVCPGLQKGQVRVEHYASKRTKFIMAHDSRLACFAL
TPDGQLLATSSNKGTLVRIFITLDGTLLQEVRRGADRAEIYSLAFSSTAQWLA
VSSDKGTVHVFNLKVNSGSSGNENTRSASDPNLVVSSSNSSLSFIRGVLPKY
FSSEWSVAQFRMLEGSQYIVAFGHQKNTVVILGMDGSFYRCEFDPALARTK
LPSAPTMVLKLHGLSVSISTARVVACLHEKSVDFELVPVDLFACENKQPEFL
AKNPFGKVPVLEDDDITLFESRAITAYVAEKFKETGHDLIRHENFNEAALVK
VWTEVESQQYNPAIDPIIFEFFAKPVVGMEPDQTVMDASLEKLKKVLDVYE
TRLSNNKFLAGDFYSLADLHHFPGTFYFMKTPWSSLVHDRPHVKAWWEEI
SSRPASKKVAEAISGSKSQHICSSVAQPSYFFSSSRVMAPVKVHGNVLSVCTR
RVIAALYEKDIKFELVPIDLGTGEHKKEPFISLNPFGEVPAFEDGDLKLFESRA
ITQYIVHEYADKGTPLVFQDSKKMAMIAVGCEVEGQKFDPAASKLTFEQVI
KPMLKMPTDAAVVEEYEAKLAVVLDVYEIRLAQSKYLAGERFTLADLHHI
PTIHYLMGTQSKKLFVSRPHVSAWVADITARPAWKKVIALQNQTQSIIDHP

HVALYWPRNGAEAAWISCVALHGSRRESRAITGYVAEKFKETGYDLITYEN

SKEAAFVKLCSLNDFNCTDGDVILGPNRSSTHQREFREAREGARRAEQQA

TNKYLAAEFYSLADLRHYPYTFYFMKTPWSSLVNDRPHVKAWWEEISARP

ASIKVAEGMKIIGELESNTDVHGNVISTAAMRVFATLYEKDIEFELVPIDMRA

GEHKKEPFISLNPFGQVPAFEDGDLKLFESRAITQYIAHEYADKGTPLVIRDS

KKMAIISLWSEVEAQKFDPAATKLTYELAIKPMFKMTTDAAVVEENEAKLA

VVLDVYETRLAQSKYLAGERFTLADLHHLPTIHYLMGTQSKKLFESRPHVR

AWVADITARPAWNKVVAQQKVMAAIKVHGNVISTAAMRVFATLYEKDIEF

ELVPIDMRAGEHKKEPFISLNPFGQVPAFEDGDLKLFESRAITQYIAHEYAD

KGTPLVIRDSKKMAIISLWSEVEAQKFDPAATKLTYELAIKPMFKMTTDAAV

VEENEAKLAVVLDVYETRLAQSKYLAGESFTLADLHHLPTIHYLMGTQSK

KLFESRPHVLAWVVDITARPAWNKLSNVRLIFCFAEIIGRQDSATAADAFCI

CFYNLFLVFEVCVPPNQ

>HF00984 MdGSTU2

MGDGDEVVLVGFWTSPYVMRVKIALAEKGVHYLYLEEEHLLQNNKSPLLL

KVNPVHQKVPVLIHNNKPVCESLIILQYIADVWSDHRPPLLSEDPYRRSKER

FWVDFFNNKIADCGRRMWACKGADQEAAKNEFVEVLKLLEGQLGEKPY

FEGDRFGLLDVALVLFACRFYTYEMFCNFSVEKECPKIVQWVKRCSLRESVS

KTLPDKYKVYDFVLEVKKMLGIN

>HF27822 MdGSTU1

MAEVKLLGSWGSPFSRRVEAALKLKNVEYEFVDEDLQSKSALLLKSNPVH

QKIPVLLHNDRPIAESQVILEYIDETWKEGFPILPKDPYERAQARFWARFIDE

KCLPATWKALWGSEEPEKAVEEACELLKILENELKDKKFFAGETVGLVDIV

ANFIAFWLRAIQELVGVELLSKEKLPKLYNWSDEFCSVFQENLPPKDRLVA

HFRVSAYSMAEEVKLLGVWGSPYSRRVEIALKLKNVEYEFVEEDLQSKSAL

LLKSNPVHQKIPVLLHNGKPLAESQVILEYIDETWKEGFPILPKDPYERAQA

RFWARFIDEKCLPATYKVLWGCEEHEKAVEEACELLKILENELKDKKFFAG

ETVGLVDIVANFIGYWLRAIQEVVGVELLTKEKLPKLYNWSDEFCSVFQESL

PPKDKLVAHIREVKLLGVWGSPFSRRVEIALKLKGVKYEYFEEDLQSKSALL

LKSNPVHQKIPVLLHNDRPIAESQVILEYIDQTWKEGFPILPKDPYERAQAR

FWARFIDEKCLPAAWKALWGSEEPEKAVEEACELLKILENELKDKKFFGGE

TVGLVDIVANFIAYWLGAIQEVVGVELLTKEKLPDLYDWSDEFCSAFHESLP

PRDKLVTFFRRRFQSTTTATSN

>HF10959 MdGSTU3

MEEVKLFGAWHSPYSSRVVWALELKGVKYEYIEEDLANKSDLLLHYNPVH

RKIPVLVHNGKPIAESSVILEYIEETWPQNPLLSNDPHERALARFWLKFIDEK

SVPFAKFFVADGEEHEKAAKEVRDLLKILEEQALGEKEFFGGSEIGLADLAL

GFIASSVGVIEQVVGVKVLQASEFPCLCNWINNFRENPAIKKSLQPADQMF

VNYKQKREMILASRP

>HF11214 MdGSTU4

MAEEVVLLDFWPSPFGMRVRIALAEKGVEYESREEDLSNKSPLLLKMNPV

HKQIPVLIHKGRPICESLIIVQYIDEVWSDKAPLLPSDPYPRAHARFWADYV

DKKIYSIGKLVWVTTGEVQEAAKKELIECFKLLENELGDKTYFGGESFGLVD

VALIPFYSWFYALETCGNMCMVKECPRLVGWAKRCMQRESVSESLPDQYK

MYDFLLELRKKFDLFCRLLILTLEPMQGSGLTMLTRRLAISVYASNCDFNDL

TILTILVYESNSDLHIYSTGKLVWVTTGSKEGTDRECFKLLENELGDKAYFRG

ESFVLVDVALIPFYSWFYALETCGNLCMVKECPRLVGWAKRCMQRESVSKS

LPDQYKMYDFLLELRKKFDSTLVSNNHPILYISLTFNNEFSNMAFVKRSKSR

KTVTFDLPEEECAEFDHGGNENQSGKEKIEGKKKGGFVRRLKKLLSKRREC

ALKESRRYDEHESKRCDKDDDNNSSVLGSNRDEDDSDNNSSVLDLHDDE

DTDCSVLDLHSDDDEDDDYSDEGLHKKAQHDHTSLMHRGGKNASQMSS

PGNRYAYPASYNTHMYPANNIYGYPDYNSAYHTYAYPSYNPDYNMHAYSA

YNANAYPAYNSASMHGAMQEYPTNNMNMPSNSVPPLDHTFNYFTEENP

ACTIM

>HF11218 MdGSTU5

MADEVVLLDFWPSPFGMRLRIALAEKGIEYEYKDEDLRNKSPLLLQMNPV

HKKIPVLIHNGKPVCESLIALQYIDEVWNDKAPLLPSDSYLRAQARFWADF

VDKKIYDIGRKLWTTKGEEYDAAKKDFLDCIGVLEGELGDKPFFGGDTLGF

VDVALIPFYSWFLVYEKFGNFSVEAVHPKFIVWVKRCMEKESVSKSLPDQEK

VYDFVALMRKKFGIE

>HF11222 MdGSTU6

MADEVVLLDSWQSPFGMRLRIALAEKGIEYEYKDEDLWNKSPLLLQMNPV

HKKIPVLIHNEKPICESLFALQYIDEVWNDRAPLLPSDSYLRAQARFWADF

VDKKKIFQIGKKVWTTKGEKQDAAKKKFLDCIGVLEGELGDKPFFGGDTL

GFVDVTLIPIYSWFLVCEKFGDFSVEAEHPKFIAWVKRCMEKESVSKSLPEQ

EKVYDYAMLIRKKLRIE

>HF11223 MdGSTU7

MADEVVLLDFWPSPFGIRLRIALAEKGIVYEYKDEDLWNKSPLLLQMNPV

HKKIRVLIHNGKPVSESLIALQYIDEVWNHKAPLLPSDSYLRAQARFWADF

VDKKMYEIGRKLWATKGEEQDAAKKEFLDCIGVLERELGDKPFFGGNTLG

FVDVTLIPYYSWFLVYEKFGNFSVEAEHPEFIAWVKRCMEKESVSKSLPEQE

KVYDYAMLIRKKLGIA

>HF11226 MdGSTU8

MADEVVLLGFWSSPFGIRLRIALAEKGIVYEYKEEDIWNKSPLLLQMNPVH

KKIPVLIHNGKPVSESLIALQYIDEVWNDKAPLLPSDSYLRAQARFWADFV

DKKIFEFGRKLWATKGEEQNAAIKEFLDFIGVLERELGDKPFFGGDTLGFVD

VTLISYYSWFLEYEKFGNFSVEAEHPEFIAWVKRCMEKESVSKSLPEQEKSLR

FFVTLETLFKSDQPSMADEVVLLDFWPSPFGMRLRIALAEKGIEYEYKDEDL

WNKSPLLLQMNPVLIHNGKPVSESLIALQAQARFWADYVHMKVIFTH

>HF11550 MdGHR1

MVSLRILRTQELQLKLLSPASTPISLFYTLFSLRTLSSYSHYDDPNSATTTAIAA

AAASSQSQSLVSSICSLVYQSYSPQTHLRSSPPKLNLDLNPESLTHEQAISVVA

SLAQEAGSMAALSFFYWAIGFPKFRYFMRLYIFCAMAILRNGNLERAHEVV

HCMVMNFAEIERLKEAADMVFEIQNQGLALSSRTLNCVLGIACDLGLVEY

AENLFEEMCDRGVSPDSLSYKSMVVGFCRNGRVSDADRWLGKMLERGFV

LDNASFTLIINMFCEKGLVGRASWCFDKMIRMGVKPNLINFTTMINGLCKR

GSIKQAFEMLEEMVRKGWKPNVYTHTALIDGLCKKGWTERAFRLFLKLVR

SDNHKPNVHTYTAMINGYCQEDKMSRAEMLLSRMKEQGLVPNTNTYTSL

VSGHCKAGNFERAYELMDIMDKEGFPPNIYTYNAVIDSLCKRGRVEEANK

LIRKGFRRGLAADRVTYTIFISEHCKRADINGALVFFTKMVKVGLQPDIHLY

TTLIAAFCRQKKMKESEKLLEFVVRADLIPTKETYTSMICGHCRDGNIASAI

KYFQRMGDHGCAPDSITYGALISGLCKEEKLEEARRLYDTMMDKGLSPCE

VTRFTLAYKYCMKDDSAAAMVMLERLEKKLWIRTVNTLVRKLCSEKKVGI

AALFFHKLVDKDQNVDRVTLAAFMTACYESNNPTCHSRVTQQLTTSTQQL

TTSTQQLKTSIFKAQRGNNTPRRLPKFPRSYISFSQSLPSDPPLEVEDRYQMA

QTTEISQSGAFLRTASVFRNFISRDPNSQFLAEPGRYHLYISYGCPWASRCLA

YLKIKGLEKAISFTSVRPTWGRTKETDEHMGWVFPASDTEVVGAEPDPLNG

AKSIRELYELASSQYTGKYTVPVLWDKKLKTIVNNESAEIIRMFNTEFNDTA

ENASLDLYPSHLQSQIDQTNEWIYDKINNGVYKCGFARKQEAYDEAVKELF

EALDKCEEILSKQRYLCGNTLSEADIRLFVTLIRFDEAYAVNFKCNKKLLREY

PNLFNYTKDIFQVPGVSSSVNMDHIKRGYYTMAAINPSGIIPIGSNIDYSSPH

DRDRSFPKYHSGNHGMDKTIRQSYQHSTRECNPDACRLPQQ

>HF11551 MdGHR2

MAQTTEISQSGAFLRTASVFRNFISRDPNSQLFLQNQGDIICIYHMVALGLL

VKPMWGRTKESDEHMGLLFPASDAEVAGAEPDPLNGAKSIRELYELASTQ

YSGKYTVPVLWDQKLKTIVNNESAEIIRMFNTEFNDMAENASMDLYPPHL

QSQIDQTNEWIYDMINNGVYKCGFARKQEPYDEAVKELFEALDKCEEILSK

QRYLCGNTLSEADIRLFVTLIRFDEAYAVNFKCNKKLLREYPNLFNYTKDIF

QVPGVSSSVNMEHIRRGYYGLLAVNPSGIVPIGPNIDYSSPHDRDSPTCHSC

VESQPPQTTKICIIATGSDLNNIFSQTLPSDPPLEDCYPMAQPMEISQSGAFV

RAPSVFRNFISRDPNSQFPAEPGRYHLYISYLCPWACRCLAYLKLKGLEKAIS

FTSVKPKWERTKESDEHMGWVFPASDTEVAGAEPDPLNGAKTIRELYDLA

STHYTGKYTVPVLWDKKLKTIVNNESSEIIRMFNTEFNDIAENASLDLYPPH

LQSQIDQTNEWIYNMINNGVYKCGFARKQEPYDEAVKELFEALDKCEEILS

KQRYLCGNTMSEADIRLFVTLIRFDEAYAVNFKCNKKLLREYPNLFNYTKDI

FQVPGVSSTVNIDHIKRGYYNIAAINPFGIIPVGPHIDYSSPHDRDRPRATAE

PPGTGSASAPFLHSRTGSELLPTLPGTPPTPSTPPIPGSGAGTPPPDPDPARSA

CDNPLARAATDRTPSISPSLSVASLLTSFVIVTFVCRSSSRIPASCVLS

>HF10119 MdGSTF2

MGVKVHGVAVSTCTSRVLTILHEKGLDFELVPVNLLAGEHKQPPFLAKNPF

GQIPVLEDGDLTLFESRAITAYVAEKFKENGVDLIRHNNLNEAALVKVWTE

VESQSFHPAISPIVFQHIIVPMRGQTPDQAVIDANLEKLAKVLDVYEDRLSST

KYLAGDFYSLADLHHLSYTYYFMKTPGASLVNERPHVKAWWEDISSRPAF

KKVAEAMTLGQK

>HF34534 MdEF1B1

MALVLHAGKTNKNGYKVLIVAEYSGVKVLAPNFEIGVTNKTPEYLKLNPIG

KYPLLITPDGPIFESNAIARYVARLKPDNPLCGSSLIDYAHIEQWIDFGSMEID

ANVLNWWLPRVGQRVYLPPAEEFAISSLKRALGALNTHLASNTYLVGHSV

TLADIVIGCNLYFGFTEILTKSFTSEFPHVERYFWTLVNQPTFKKVIGDVKQA

VSVPPVVSAKKPAQPAKGKAKEEPKKEAKKEPEKPKAEAAEEVEEAPKPK

PKNPLDLLPPSKMVLDDWKRLYSNTKTNFREVAIKGFWDMYDPEGYSLWF

CEYKYNDENTVSFVTLNKVGGFLQRMDLARKYAFGKMLVIGSEAPFKVKG

LWLFRGQEIPKFVMDECYDMELYNWTKVDISDENQKERVNQMIEDQEPFE

GEALLDAKCFK

>HF35045 MdGSTU9

MAQYTPTLNPPKTLIPKLSNPNPSFSSFSTFTIPKPKSINFPKFSISVTAAQNQ

QHRQQPPSLEALINSDRKEEVLGVIKSSSSHCLSETNLHLTVPGLKSKTRGK

VRDVYDSGDYLVMVTTDRQSAFDRILASIPFKGQVLNETSLWWFDKTRHIT

SNAVVLAPDENVTIAKKCSVFPVEFVVRGFVTGSTDTSLWTVYKNGVRNY

CGNVLPDGLVKSQKLPANILTPTTKAADHDVPVTPDEIIERGLMTQAEYDE

VSRKALSLFEYGQRVALEHGLILADTKYEFGKGQDGSILLIDEVHTPDSSRY

WIAHTYEERFQNGLEPENVDKEFLRLWFKDHCDPYEDEVLPDAPEELVCE

LAWRYIFLYETITKSKFKLPSTEEPIHDRISRNVAQALCLTSGLYATLVKLLGY

WASPFALRVKWALKLKEIEYQYVEEDLPNKSPLLLRYNPVHKKVPVLVHD

GKPVAESLIILEYIDETWKHNPILPQDPYERAQARFWARFIDEKCVPAIMSAF

TNQGEEKEKAAKEARENLKTLESALAGKDFFGGETVGFLDIAAGWIGLWA

RIVEEIADVNLIDTETMPLLNAWFGRVLEAPVLKECVPPQDKLLEHNRGCK

ASAFAVIPFHICFQGHWRLEYHT

>HF34035 MdGSTF3

MHLSHRGIGHLCRYALRFLDRSISRAMGCFFGCFRVKDDRHLRCRPHLVSQ

SSRNPQTEVVISQNRLSSLFSAEEKEGSPSKDWESASYGTPQIDKELRKEAKF

LKACGTIAETPAEIRKVSSTLNGSPHRDRDSEPPKFNSQLPKTSIKNLQQELD

QPDQPPTPMKFNEEWGKGSGPSEHTPSSFVSNQQNNGRTTISSSGGSGIGT

DSTVKSHTNRTENITTPDVQFRGKAVRFDCNNDTNSSRGSSSENYNQSSKV

CETPGKQSISKPSVYPTPLTLSDEMQTPGTVFPANLENFPNGKARIRSQYVYS

VQNPGDGISQQNVLKDEDPNSHGLSVEQRESLNHWGNATPISEEGAKQIS

SGQEVNVESSLSTWLKPVTQDNNNQFGNVPGRTRHFGRTPGDRPIIGLVAT

HWNEDEPTRVSPKWWDGNGIPNSTNKYKEDQKVSWHATPFEERLEKALS

EESFISQRKNIEGKPIVFDENDESDTALSQLQSSSQPKSVQFSTTFAMVVKVY

GPFYALPKRVLVCLVEKEIEFETSPIDLFKGEHKTPEFLELQPFGQVPVIQDG

DYTLYESRAIIRYYAEKYSSQGTDLLGKTIEERGLVEQWLEVEASNYHPPLD

NLVINILFASALGFPSDPKIIQESEEKLGKVLDIYEDRLSKSKYLAGDFFSLAD

LSHLPFTHYLANSMGKEYMIRDRKHVSAWWDDISSRTSWKKEATNGWMY

AYDVSSPTYPPVSTPILTSSTPIVGVSLVPHHALPI

>HF34036 MdGSTF4

MVVKVYGPLYASPKRVLVCLVEKEVDFETVPIDLLKGEHKHPDFLKLQPFG

SVPLIQDGDYTLYDRKIIQESEEKLGKVLDIYEERLSKSKYLAGDFFSLADLS

HLPFTHYLVANMGKEYMIRDRKHVSAWWDDISNRPSWKRVYGPVYPSPK

RVLVCLVEKEIEFETVPIDIFKGEQRNPEFLKLQPFGQVPVIQDGDYTLFESR

AIIRYYAEKYKSQGTDLLGKTMEERGLVEQWLEVEASNYHQPLDNIVTHIL

FASAMGISSDPKIIQESEEKVGKVLDIYEERLSKSKYLAGDFFSLADLSHLPFT

HYLANSMGKEYMIRDRKHVSVWWDDISSRPSWKKVLQFGECNECICLPR

NIAT

>HF36976 MdGSTU10

MSTEDVVLLDCWVSPFCMRVKIVLEEKGIQYEERAEDLFGGKSELLLTSNPI

YTKVPVLLHKGKPVCESSIIVGYVDETWASPPLLPSCPYARAQAKFQADYID

KKLYDAGSSIWRTKGEAQEVAVKDFIEVLKQLEKFLGDKDFFGGDSFGFVDI

ITIGITSWFSAYEKFGNFKVEDHTPKLSAWINRCMQRKTVAKVIPDPEKVYE

FVINLKKMMGFE

>HF22792 MdGSTU12

MADEVVLLDFWPSPFGMRLRIALAEKGVEYEYKDEDVRNKSPLLLQMNP

VHKKIPVLIHNGKPVCESLIALQYIDEVWNDKAPLLPSDCYLRAQARFWA

DYVDKKIYEFGRKLWTTKGEEQDAAKKEFLDCMGVLEGELGDKPFFGGET

IGFMDVALIPFYSWFLLYEKFGNFSVEALHPKFIAWVKRCMEKESVSKSLPE

QEKVYDFVMLMRKKLGME

>HF22793 MdGSTU13

MADEVVLLDFWASMFGMRARVALAEKGVKYEYREEDLWNKSQLLLQMN

PVHKKIPVLIHKGKPVCESLIIVQYVDEVWRDKAPLLPSEPYQRAHSRFWA

DLIDKKLYDAGRKIWSTKGEEQEAAKKDFIEILKQLEGQLGDEPYFEGEKFG

FLDIVLITFYCWFHTYETCGNFSIEAECPKLIAWAKRCMQRESVSKSLADEK

KVMFGMRARVALAEKGVKYEYREEDLRNKSQLLLQMNPVHKKIPVLIHN

GKPVCKSLIIVQYIDEVWRDKAPLLPSEPYQRAHSRFWADLIDKKLYDTGR

KIWSTKGEEQEEAKKDFIEILKHIEAECPKLIAWAKRCMQRESVSKSLADEK

VYEFVLGMKKMLVCSSTMADEIVVVSFWTSMYGFNTASECHSSFVIRTRGF

GILDQHMFGVRARIALEEKRRGLVEQEPSPSADESSSQEDPGSHPQWQTVI

DEVWKDQAAPFLPLDPYNRARARFWADLIVSCCWEEAMDHKGEEHEAA

KKEFIEILKQLEGELGDKPYFGGERFGFLDIALIGFYNYFYSRGHLSMEAECP

KIIAWANRCLLRESVSKSIADPKKVCELLLRFLA

>HF22794 MdGSTU14

MNPVHKKIPVLIHNGKPVCESLIIVQYIDEVWRDKAPLLPSEPYQKAHSRF

WADFIDKKLYDAGSKIWLTKGEEQEAAKKDFIEILKQLEGQLGDKPYFEGE

KFGFLDIALITFYCWFHAYETCGNFSIEAECPKLIAWAKRCMQRESVSKSLA

DEKKVYELVLGVQKMLGVD

>HF22797 MdGSTU15

MADEVVLLDYWASMFGMRARVALAEKGAKYEYREEDLRNKIKSQVLLQM

NPVHKKIPVLIHNGKPVCESLIIVQYIDEIWRDKAPLLPSEPYQKAHSRFWA

DFIDKKLYDAGSKIWLTKGEEQEAAKKDFIEILKQLEGQLGDKPYFEGEKFG

FLDIALITFYCWFHAYETCGNFSIEAECPKLIAWAKRCMQRESVSKSLADEK

KVYELVLGVQKMLGVD

>HF22798 MdGSTU16

MADEVVLLDYWASKFGMRARVALAEKGVKYEYREEDLRNNIKSQMLLQ

MNPVHKKTPVLIHNGKPVCESLVILQYIDEVWRDKAPLLPSEPHQKAHSRF

WADFIDKKLYDACTKIWMTKGEEQEAAKKDFIEILKQLEGQLGDKPYFEGE

KFGFLDIALITFYCWFHAYEICGNFSIEAECPKLIAWAKRCMQRESVSKSLA

DEKKVYELVLGLQKMFGWE

>HF22799 MdGSTU17

MADEVVLLDFWASMFGMRARVALAEKGIEYEYREEDLRNKSQLLLQMNP

VHKKIPVLIHNGKPVCESLIIVQYIDEVWSDKAPLLPSEPYQRACSRFWADFI

DKKLYDAGRKIWSTKGEEQEAAKKDFIEILKQLEGQLGGKPYFEGEKFGFL

DIALITFYSWFHAYETCGNFSIEAECPKLIAWAKRCKQRESVSKSLADEKKV

YEFVLSIKKMLGVE

>HF23084 MdGHR3

MSILGNGILDHEVVHCMVMNFAEIERLKEAADMVFEMQNQGLALSTRTL

NCVLGIACDLGLVEYAENLFEEMCERGVSPDSLSYKSMVVGFCRSGRVSEA

DRWLSKMLERGFVLDNSSFTLIINMFCEKGLVGRASWCFDKMIRMGVKPN

LINFTTMINGLCKRGSIKQAFEMLEEIVLSYCKKGWTERVFRLFLKLVWSDT

HKPTVHTYTAMISGYCREDKMSHAEMLLSRMKEQGLVPNTNTYTTLISGN

CRAGNFERAYEFMDIMGKEGFPPNICTYNVVNDSLCKKGRVEEANQLIKG

GLRQGLAADGITYTIFISEHCKRADINRALVFFTKMVKVGLQPDIHLYRTLI

AAFCRQKKMKESEKLFEFAVRAGLIPTKETYMAMICGHCRDGNIASAVMY

FQRMGDHGCAPDSIIYGALMSGLCKDEKLEEARRLYDPMMDKGLSPCEVT

RLTLAYKYCMKDDSAAAMVMLERLEKKLWIHTVNTLVRKLSSDKKVGIAA

LFFHKLVDNDQNVDRVTLADTDDLTSIVILGLRQQRRVLFTAHSVQHSSIA

VLVPSPMSGRSRDYIGDRPNFGLAADEARSKGYKLEAVEATWHPCNNKQD

VKSDKGKDNSGASDDNSRRRLLSALDSSGGVADIADYRYKSGQRLEENEA

QDCDEGTDELPEMPNEGAQNCYYSRWYLCVNSVALGEEKDRVVVIGEGV

DAFKLAKSLRKKFKAADIITTSFSICLLLALKSLVGRTQSSPTALHHLSLSLAL

SRSLCPSLSLRSPSSLQDVLLQQLDKILDVLISAGIHSSAHNQMARSALDKTS

LSGAFVRTASVFRNFISRDPNSQFPAEPGRYHLYISYACPWASRCLAYLNIKG

LDKAISFTSVKPIWERTKERDEHMGWVFPASETELAGAEPDPLNGAKSIREL

YELASTQYTGKYTVPVCSLILSGLFIFLSLTILYQMTDRFTLTNEWIYDKINNG

VYKCGFARKQEPYDEVYGVHFKCNEKVLREYPNLFNYTKDIFQIADHRYM

SGQKDSKKMKRKIVMKVPMNCRKCQAKALKIAATVDGVSSVALGEEKDR

VVVTGEGVDAIKLAKSLRKKFKAADIITTLPFLFVYCYHSESGWSDSELTNS

TTSPVSLSCSLSPSLALRSPSSLQDVLLQQLDKILDVLISAGIHSSAHNQMAR

SALDETSLSGAFVRTASVFRNFISRDPNSQFPAEPGRYHLYISYACPWASRCL

AYLNIKGLDKAISFTSVKPIWERTKESDEHMGWVFPASETELAGAEPDPLN

GAKSIRELYELASTQYTGKYTVPVLWDKKLKTIVSNESGEIIRMFNTEFNDIA

ENASLDLYPPHLQSQIDQTNEWIYDKINNGVYKCGFARKQEPYDEAVKQL

YEALDKCEEILSKQRYLCGNTLSEADIRLFVTLIRFDEVYVVHFKCNKKVLR

EYPNLFNYTKDIFQVPGMSSTVNMDHIKRHYYGSHPSINPFGIIPSGPDIDYS

SPHDRNRFST

>HF29919 MdGSTU11

MTSTFVACFQSVGEEHEKAKQETQVVFKTIEHGLGDKKFFGGRQLVWQT

WHLGWIAGWRGATEESAGLKLLEPNIFPHLHTWISSSKDVPVVKENLHYK

KTQQIRNLNTKITKMAQVKLLGLWPSPYVYRVIWALKLKGVEYEYVEEDVL

FNKSDELLKYNPVHKKVPVLVHDGKATAESIVILEYIEEAWPHNPLLPKDP

HQRAEARFWTKFGEDKNRAFLGFFFATGEEQVKAVKEAQENLKILEEHGL

GNKKFFGGDKIGLVDLAFGWIALWLEVLEESAGVKVFEADSFPRLHAWIQS

FKESSTIKENLPDRDRSAFLNYFKGRRATIEPQHKIRKMAEVKLLGAWPSPF

VQRVIWALKLKGVEFENVEEDVLFNKSDELLKYNPVHKKIPVFVHNGKPV

AESIVILEYIDEAWPHNPLLPKAPHLRAEARFWAKFGEDKDRAFRGFFFVT

GEEQVKAVKESQENLKILEEHGLGNKKFFGGEAIGLVDLAFGWIACWLEV

WEEVAGVKVFEADRFPRLQAWIQRFREVPTIKENLPDRTALLTFASSLNPV

HKKVPVFAQGGETIGESIYIERAWPQNPLLPNDPHVVESCPTSVRNKVCYV

LIWSWATLHIANWFYGEIPISSWYQSKVVHPNGHTRSMSLSCCPRVDLKIR

HTCRDVLRVVPHRGLSSCEAKRPHALYVTQLLSTCRLENPPKVRGRVKSCP

TSVRDKVFYVLIWSWATLHITNWFYGGIPISSHARAVARFWTKFGDDKKAN

FLDFYKGAGEEQARAAKDAQEHLRILEERGLGMMDLSFGWIAFWLEAKE

EAAGVQVLEVDSVPRLEA

>HF28390 MdGSTF5

MATLSALATTWPDPNSSAAFLSDPTDDNPTSVVDSSDDNHDPQSPPNPNS

DHNLHNNIPLSSSPPESAPPAPRSPPSLFHVCFNQDQACFAVGTDHGFRIYN

CDPFRELFRRDFDNGGGIGVVEMLFRCNILAIVGGGPDPQYPTNKVMIWD

DHQGRCIGELSFRSVIRSVRLRRDRIVVVLEQKIFVYNFADLKLLHQIETIAN

PKGLCAVSQVAGSLVLVCPGLQKGQVRVEHYGSKRTKFIMAHDSRLACFAL

TPDGQLLATASNKGTLVRIFNTLDGTLLQEVRRGADRAEIYSLAFSSTAQWL

AVSSDKGTVHVFNLKVNSGSSNENPRSASDPNLAVSSSNSSLSFIRGVLPKY

FSSEWSVAQFRLLEGSQYIVAFGHQKNTVVILGMDGSFYRCEFDPVNGARV

VACLHEKSVDFELVPVDLFACENKQPEFLAKNPFGLVPVLEDDGITLFESRA

ITAYVAEKFKETGHDLVRRENFNEAALVKVWTEVESQQYNPAIEPIIFEFFAK

PVVGIEPDQTVIDASLEKLKKVLDVYEARLSSNKFLAGDFYSLADLHHFPG

TFYFMKTPWSSLVDDRPLVKAWWEEISARPASKKVAEETRAIRQYIAHEYA

DKGTPLVIRDSKKMAILSLWTEVEGQKFDPASSKLTYELAIKPLLKMTTDAA

VVEECEPKLAVVLDVYETCLAQSKYLAGKSFTLADLQPLPTIHNLMGTQSK

KMFESRPHVSAWVAYITARNLISTDFN

>HF04750 MdGSTL1

MMGTASVIHNGVHIHLKLNSRLPRLSSPYPSPNWSRPHFVHNHIVSVRTEE

KRGDSASCDGISNVEISHDDSPEKIKGREAYAASAKTVALWVCAAVAFGIG

VGFKDGVGKATEFISGMYRVFTGTKLVSRQAVCLCSSFQLLQSATYVSATYD

GNRFFTFQDGVRKATPLLLTVAVIELSDVAFAVDFPTYQHEELPPTLDSTSSP

PPLFDGTTRLYVNYSCPFAQRVWITRNYKGLQDKIKLVPINLQNRPDWYKE

KVYPGNKVPALEHNGKVIGESLDLIKYIDSNFEGPSLFPKDDPERKKFGEEL

LTYVDTFTGSLYRSFKGDAVKAADEQFDYLENALKKFDDGPYFLGQFSLVD

IAYIPFIERFQVFLSDVFKYDITAGRPKLAAWFEEINKIEAYKVTKTDPKELVA

FYKKRFLEQQ

>HF06446 MdGSTU18

MGGTGCSTGQILGVFVFHLPPWNGFVPRMWNTMFYHFKTNIPHVFFKNY

TFVPSRPVPSRSVPSRPVPSTYQTHPYGSIHGLPCYNQKTPTYMQGLQPSTR

RYAHLVSQIITNVNIILQRAPSFNEVMLLGVWGSPFSRVEIALKLKGVKYEY

VEEDLPNKTLRLRGVKAESYEEDLQNKSALLLKYNPVHKKVPVLVHNEKPI

AESLVIVEYIDETWKHQGSPILPEDPYQRANARFWARFIDEKCKPGIIKAFFE

KEERLKAVDEARGLLKLLENELKDNKFFGGETIGFVDIAANLIGHWLKPAQ

EALELGLLSKEKSPKLWDWCDEFVSVVKETLPSTDNLIGLILSRASST

>HF42508 MdGSTU19

WVQWFPSLRGVAAAEGDEARKAAVEQVAEGLDKMEEAFQKTSKGKDFFS

GDKIGYLDIAFGCFLGWLRVTEKMNGIKLLDEAKIPGLAKWAEKFSADPAV

KDVMPETDKLAEVAKIVTARLRAAGASNRGLIMGESDVKVLGMAPSPFV

MRARIALNLKSVDYEFLQETFGSKSELLLQSNPIHKKVPVLIHGDKPVCESL
VIVEYIDEVWASGPSILPSDPYDRATARFWAAYISEKWYPSMKGIGFAQGEE
AKKAAIEQVTEGLALLEEAFEKSSKGNVFFGGDEIGYLDIAFGCFLGWLRV
NEKLHGIKLLDQTKTPGLVKWADKFCAHAAVKDVMPETDMLVEIAKILA
AKARAAAAPPPTSSDACVSAINAFYDQVGFKLSAAAAHDDQCVLANILA
AYFLSSDPSRAPSHLQAAKSRLEQATPYEKAVFDAVNCLISKDRDDDVAFEL
HSKLLKSFPRDLASLQRAQVLCFYMARPDLSLDLVQQTMLNAVEDNFLAVI
FRASCMSIEINKCDCWVQHNMCHVLQYNCRFKEAAELMEECSPSWDLCS
SFMFTHNWWHVALCYLEGHAPIQRIRDLYDHFIWKELEKPDAPRPEVYLN
ALGLLLRVHARGEMDAFKDHLKTVENCVTDQANWYLDWQFDMLVLWA
LAYTGEISKAEELLKGLKPRITKMNEKKQQFMQNAILLTEATYEYGKGNGK
QALELLGPDFDADKLQAIEVLKKRIKTREGIPFLWRLLERGYKLTGRDEEAA
TASEEAKRLEKAYFP

>HF24574 MdGSTU20

MGVVKLFATEGSVFCARIQWALKLKGVEYEFILEDLANKSPLLLKYNPIYKK
VPVLVHGDNVIVESLVILEYIEDTWKEHPLLPRDPYDRATARFWAKFNDEK
LLLSVWAACTGEGEAQEKAKESALESLALVEKQIEGKKFFGGEQIGYLDLV
LGWIPHWISAMEEAGGNKVPEAEMFPSLHQWGQNFIHTPLIEECIPAKEAL
VPYLRFMIDFKMGDVKVIGASLSLFCCRIEWALKHKGIAYEYIEEDLRNKSH
LLLRYNPVHKKVPVLVHGEKPVAESLVILEYIDETWRENPLLPEDPFEKATA
RFWAKYVDEKCVISAWTASRTKGHEQEKSLEAARESLEVLNKLIEGKKFFG
GEIIGFLDLVVGTLPNWLKFLEESEGIKLFDTKELPFLHEWAQRFTEIPIIKGS

MPTAEDLINYNRDRQKAE

>HF12597 MdTCHQD1

MQLYHHPYSLDSQRVRLALEEKGIDYTSFHVNPVTVKNMDASFFRRNPSA

KLPVFQNGHHIIYNTIEIIQYVERIALVSSGEENIPSSGGEVTAWMHRIKQWN

PKFFTLSHIPKKHRDYVYKFIRQVIIARMAEATDLAAAYHTKLVQVYETEDK

LKDPAVLKQSKEQLIRLLDQVEKRLNESAYLAGEEFSMADVMLVPVLARLV

LLNLKEEYIDGRPNTAKYWIMVQQRPSYKKVIGKHFSGWRKHKTFIRTWC

FVHIRTILRRF

>HF17033 MdEF1B3

MKRLLVLMEKSRVAGRCHNQPWFFLLIASFLLVFVLLLSDYSALSAGRNTQ

QVTYFITNFANVIVNQDNSTSLPYHDHSTPISSNQTEIWLTLNVTNSSTSNAI

PAQFFGHGREENHSVKSVVISKSPPAQPISPPPVQSISSPPVQSISSPPAQPISN

EIGKNNSDADSCSGRYIYVYDLPKRFNQDLLKNCHSLVRWNDMCSHLSN

MGLGPRIHNSKGKLVGNGWFATNQFSLEVIFHNRMKKYRCLTNDSSLASA

IFVPFYAGLDVGRYLWDYNTSVRDASPLELVKWLSRRPEWKAMWGRDHFL

VGGRIAWDFRRLTDNNSDWGSKLMFLPESKNMTLLSIESGSWNNEQAIPY

PTYFHPSKDSEVFEWQRRMRNRKRRYLFSFAGAPRSDSKDNIRDTIINQCRS

SATCKLVSCYNGAKKCDDPLNVMKVFEASVFCLQPSGDSYTRRSTFDSILA

GCIPVFFHPGSAYVQYLWHFPNTPSKYSVFISENDIKDQKAIINETLLRIPKH

QVVAMRKEVVRLIPRVIYANPMAPRLETVEDAFDIAVKGMLDKVEKIRRD

MKEGKDPGVAFSELNGRKFDMPAVYRLLLSNFSLGRTLRLTDLKSERNVFL

EQPWLCVDLVDDDTVLHAGKTNKNGYKALITAEYTGVKVELAPNFDMG

VSNKTPEYLKLNPIGKVPLLVTPDGPIFESNAIARYVARLKADNPLYGCSLID

YAHIEQWIDFGSMEIDANISKWYYPRLGYGVYLPPAEEAAISALKRALGAL

NTHIASNTYLVGHSVTLADIVVVCNLYVGFANVMTKSFTSEFPHVERYFWT

LVNQPNFKKVLGDVEQAVSVPPVASAKKPAQPAKGKIKEEPKKEAKKEPA

KPKAEAAEEVEEAPKPKPKNPLDLLPPSKMVLDDWKRLYSNTKSNFREVAI

KGFWDMYDPEGYSLWFCEYKYNDENTVSFVTLNKVGGFLQRMDLARKYA

FGKMLVIGSEAPFKVKGLWLFRGQEIPKFVMYECYDMELYSWTKVDISDEN

QKERVNQMIEDQEPFEGEALLDAKCFK

>HF17304 MdEF1B2

METRLVLHAGSTNKNGFKALIAAEFSGVKVELVKNFEMGVSNKSPKFLEM

NPIGKVPVLETPDGPVFESNAIARYVTRLKADNPLYGSSLIEYARVEQWIDF

ATLEIDANILRWLIPRIGFSVYLPPVEEAAVSALKRALTALNTHLSSNTYLVG

HSVTLADIILTCNLTLGFSRLFTKSFTSEFPHVERYFWTLVNQPNFRKILGEV

KQAESVPSIQSVKKPSQAKEPAKPKAKDEPKKGDKKESAKPKLEEAAEEEA

PKPKAKNPLDLLPPSKMILDDWKRLYSNTKTNFRQVAVKGFWDMYDPEG

YSLWFCDYKYNDENTVSFVTLNKVGGFLQRMDLARKYAFGKMLVIGSAPP

FKVKGLWLFRGQEIPKFIIDECYDMDLYEWRKVDITDEDQKERVNQMIEDH

EPFEGEDLLDAKCFK

>HF07438 MdGSTU21

MAKSNVKVLGAWPSPYVMRARVALNIKSVEYEFLEETFGSKSQLLLQSNPV

HKKIPVLIHGDKPVCESLIIVEYIDEVWSSGPSILPSDPFDRATARFWAAHIDE

KWFPAMKGIGAAQGDDARKAAVEQVAEGLAQLEEAFQKTSKGKDFFSGD

KIGYLDIAFGCFLGWLRVTEKMNGVKLLDGAKIPGLVTWAEKFSADPAVK

DVMPETDKLAEFAKILAAKMRGAGAPK

>HF29092 MdGSTF7

MGLCTAPQSVCWCALLRKKEVDFETVSIDVLKGEHKHPDFLNHLDQFLSF

KLGIIYTLYESRAIIRYYAEKYKSQGTNLLGKTIEERGLVEQWLEVEAHNFHP

PSYNMVLHILFASVSGFPSDPKIIQESEEKLGKVLDIYEERLSKSKYLAGDFFS

LVDLSHLPLTQYLVANMGKEYMIRDRKHVSAWWDDISNRPSWKRMLLRH

KKTTNGWMYTCEQPHPPSTPILNSSIQLLECYIIRSKEPEAGVCRSDLNPSVL

TLRSHNMVVKVYGPLYAAPKRVLVCLVEKEVHFETVPIDLLKGENKHPDFL

KLQPFGTVPLIQDGDYTLYESRAIMRYYAEKYKSQGTDLLGKTIEERGLVEQ

WLEVEAHNFQPPIYNLVVHILFAPVLGFPSDPKILQESEEKLGKVLDIYEEQL

SKSKYLAGDFFSLADISHLPFTHFLVANMGKEYMIKDRKHVSAWWDDISN

RPSWKRVLQFGDPF

>HF29096 MdGSTF6

MRRICFPRFVCSKFKNDPTTPIPLSLSLSTESINHLKRKTKNKLEFWISPLFLL

FLFVLKSMLCSLLKRGLGNLCRSALRFLDRSISRAMGCFFGCFRDKDDRHL

RSRPHLVSQSSRNPQTEVVLSQNRLSALFSAEEQEGSPSKDRESPRYGSPQID

KELRNEAKFLKACGTIAETPAEIRKVSSRLNGSPHHDRDSEPPKFNSWLPNT

SFRNLQQELDQPDHPLTPMKYNEEWGMGSGPSEHTPSSCEFNQQNTGRIA

ISSSGGSGIGTDSTVKSHTNRTENITTPDVQCRSKAVRFECDNNTNSSRGSSS

ENYNRSSKVCETPGKQSISKPSVYPTPLTLSEEMQTPGTVFPANLENFPNGR

ARIRSQYVYSVQNPVDGISQWNVLKDEDPKSLGHSGEPRESLNDWGNATP

TSEEGSKQMSSGQEVNVESSLSAWLKPVTQDNNNHFGYVPGQTRHFGRTP

VDRPIIGLVATHWNEDEHTRVSPKWWDGNGIPNSTNKYKEDQKVSWHAT

PFEERLEKALSEESFISQRKNMEGKPIVFDENDESDTALSQLQTSSHPKSVVY

GPFYAAPKRVLVCLVEKEIEFETSPIDLFKGEHKSPEFLKLQPFGQIPVIQDGD

YTLYESRAIIRYYAEKYKSQGTDLLGKTIEERGLVEQWLEVEASNYHPPLYNL

VMHILFASGLGFPSDPKIIQESEEKLGKVLDIYEERLSKSKYLAGDFFSLADLS

HLPLTQYLVASMGKEYMIRDRKHVSAWWDDISSRPSWKKVYGPFYASPKR

VLVCLVEKEIEFETSPIDLFKGEHKSPEFLKLQPFGQVPVIQDGDYTLYESRAII

RYYAEKYKSQGTDLLGKTIEERGLVEQWLEVEASNYHPPLDNLVMHILFAS

ALGFPSDPKIIQESEEKLGKVLDIYEERLSKSKYLAGDFFSLADLSHLPFTHYL

ANSMGKEYMIRDRKHQTILEEGPPIWCAVLRYLKKLLHSQHCHYKSQGTN

LLGKKIEERGLVEQWLEVEAHNFHPPSYNMVLHILFASVLGFPSDPKIIQES

EEKLGRVLDIYEERLSKSKYLAGDFFSLADFSHLPLTQFLVANMGKEYMIKD

RKHVSALWDDISNIPSWKRVLQFAPPTLHSNFKFFHQLLGCYIIRSKEPQAG

VCRLNLNPSVLTLRSHNMVVKVYGPLYAAPKRVLVCLVEKEVDFETVPIDL

LKGENKHPDFLKLQPFGTVPLIQDGDYTLYESRAIMRYYAEKYKSQGTDLL

GKTIEERGLVEQWLEVEAHNFQPPIYNLVVHILFSPVLGFPSDPKILQESEEK

LGKVLDIYEEQLSKCKYLAGDFFSLADLSHLPFTHFLVANMGKEYMIRDRK

HVSAWWDDISNRPSWKRVLQFGDPF

>HF32109 MdGSTF8

MVVKVYGPVMAACPQRVMVCLLEKGVNFEIVDVDLEAGEHKRPEFLTRQ

PFGQVPVVEDGDFRLFESRAIIRYYAAKYAGRGPNLLGTTLEEKAVVDQWL

EVEGHNFNDLVYTLVLQLLVLPRMGQRGDAALINACEEKLEKVFDVYEER

LSKSKYLAGETFTLADLSHLPGIRYLIDEAKLGHLVTGRKKVNAWWEDISN

RPSWKKLMQLASDY

>HF32118 MdGSTU22

MATEDVVLLDCWASPYCMRVKIALEEKGIKYEERAEDLFGGKSELLLTSNP

VYAKVPVLLHKGKPLCESSIIVGYIDETWASPPLLPSCPYARAQAKFWVDYV

DKKLFDAGGNIWRTKGEAQEVAVKDFIEILKQLEKVLGDKDFFGGDSFGFV

DIITIAITSWFLAFEKFGNFKVEDHAPKLSAWIKRSMQRETVAKVIPDPEKIY

EFVINLKKMMGME

>AtGSTU1 XP_020885836.1

MTEKEESVKLLGFWASPFSRRVEMALKLKGVPYEYLEEDLPNKTPLLLELN

PLHKKVPVLVHNDKILLESHLILEYIDQTWKNNPILPQDPYEKAMARFWA

KFIDEQILTLGFRSLVKAEKGREVAIEETREMLTFLEKEVTGKDFFGGKTIGFL

DMIAGSMIPFCLARLWEGIGIDMIPGEKFPELNRWIKNLEEVEAVRGCIPPR

DKQIERMTKIAETIKSA

>AtGSTU2 NP_180509.1

MAKKEESVKLLGFWISPFSRRVEMALKLKGVPYEYLEEDLPKKSTLLLELNP

VHKKVPVLVHNDKLLSESHVILEYIDQTWNNNPILPHDPYEKAMVRFWAK

FVDEQILPVGFMPLVKAEKGIDVAIEEIREMLMFLEKEVTGKDFFGGKTIGFL

DMVAGSMIPFCLARAWECLGIDMTPEDTFPELNRWIKNLNEVEIVRECIPP

KEKHIERMKKIIERAKSTF

>AtGSTU3 NP_180508.1

MAEKEEGVKLIGSWASPFSRRVEMALKLKGVPYDYLDEDYLVVKSPLLLQL

NPVYKKVPVLVHNGKILPESQLILEYIDQTWTNNPILPQSPYDKAMARFWA

KFVDEQVTMIGLRSLVKSEKRIDVAIEEVQELIMLLENQITGKKLFGGETIGF

LDMVVGSMIPFCLARAWEGMGIDMIPEEKFPELNRWIKNLKEIEIVRECIPD

REKHIEHMMKIVGRIKAV

>AtGSTU4 NP_180507.1

MAEKEEDVKLLGFWASPFTRRVEMAFKLKGVPYEYLEQDIVNKSPLLLQIN

PVYKKVPVLVYKGKILSESHVILEYIDQIWKNNPILPQDPYEKAMALFWAKF

VDEQVGPVAFMSVAKAEKGVEVAIKEAQELFMFLEKEVTGKDFFGGKTIGF

LDLVAGSMIPFCLARGWEGMGIDMIPEEKFPELNRWIKNLKEIEIVRECIPPR

EEQIEHMKKVVERIKSA

>AtGSTU5 NP_180506.1

MAEKEEVKLLGIWASPFSRRVEMALKLKGIPYEYVEEILENKSPLLLALNPI

HKKVPVLVHNGKTILESHVILEYIDETWPQNPILPQDPYERSKARFFAKLVD

EQIMNVGFISMARADEKGREVLAEQVRELIMYLEKELVGKDYFGGKTVGF

LDFVAGSLIPFCLERGWEGIGLEVITEEKFPEFKRWVRNLEKVEIVKDCVPPR

EEHVEHMNYMAERVRSS

>AtGSTU6 NP_180505.1

MGKNEEVKLLGIWASPFSRRIEMALKLKGVPYEYLEEDLENKSSLLLALSPI

HKKIPVLVHNGKTIIESHVILEYIDETWKHNPILPQDPFQRSKARVLAKLVD

EKIVNVGFASLAKTEKGREVLIEQTRELIMCLEKELAGKDYFGGKTVGFLDF

VAGSMIPFCLERAWEGMGVEMITEKKFPEYNKWVKKLKEVEIVVDCIPLRE

KHIEHMNNMAEKIRSA

>AtGSTU7 NP_180503.1

MAERSNSEEVKLLGMWASPFSRRIEIALTLKGVSYEFLEQDITNKSSLLLQLN

PVHKMIPVLVHNGKPISESLVILEYIDETWRDNPILPQDPYERTMARFWSKF

VDEQIYVTAMKVVGKTGKERDAVVEATRDLLMFLEKELVGKDFLGGKSLG

FVDIVATLVAFWLMRTEEIVGVKVVPVEKFPEIHRWVKNLLGNDVIKKCIPP

EDEHLKYIRARMEKLNIKSA

>AtGSTU8 NP_187538.1

MNQEEHVKLLGLWGSPFSKRVEMVLKLKGIPYEYIEEDVYGNRSPMLLKY

NPIHKKVPVLIHNGRSIAESLVIVEYIEDTWKTTHTILPQDPYERAMARFWA

KYVDEKVMLAVKKACWGPESEREKEVKEAYEGLKCLEKELGDKLFFGGET

IGFVDIAADFIGYWLGIFQEASGVTIMTAEEFPKLQRWSEDFVGNNFIKEVL

PPKEKLVAVLKAMFGSVTSN

>AtGSTU9 XP_020869516.1

MDEEIENKVILHGSYASPYSKRIELALKLKSIPYQFVQEDLQSKSQTLLRYNP

VHKKIPVLVHNGKPISESLFIIEYIDETWRNGPHLLPEDPYRRSKVRFWANYI

QLHLYDVVIKVVKSEGEEQERALTEVKEKLRVIEKEGLKEIFSDTDGEPTVT

NETMSLVDIVMCTLLSPYKAHEEVLGLKIIDPEIVPGVYGWINAINETRVVK

DLSLPYEQVLEILRVFRQMSLSRS

>AtGSTU10 NP_177598.1

MEEKKSKVILHGTWISTYSKRVEIALKLKGVLYEYLEEDLQNKSESLIQLNPV

HKKIPVLVHDGKPVAESLVILEYIDETWTNSPRFFPEDPYERAQVRFWVSYI

NQQVFEVMGQVMSQEGEAQAKSVEEARKRFKVLDEGLKKHFPNKNIRRN

DDVGLLEITIIATLGGYKAHREAIGVDIIGPVNTPTLYNWIERLQDLSVIKEV

EVPHDTLVTFIQKYRQKCLQQAANA

>AtGSTU11 NP_177151.1

MGLMNRSKNDEYVKLLGAWPSPFVLRTRIALNLKNVAYEYLEEEDTLSSES

VLNYNPVHKQIPILIHGNKPIRESLNIVMYVDETWLSGPPILPSDPFDRAVAR

FWDVYIDEHCFTSINGVAVAKGEENINAAIAKLEQCMALLEETFQECSKGR

GFFGGENIGFIDIGFGSMLGPLTVLEKFTGVKFIHPENTPGLFHWADRFYAH

EAVKPVMPDIEKLVQFARLKFNTSIFK

>AtGSTU12 NP_177150.2

MLKNKKSDNSLSRDTLQIKKRKKTTMAQNGSNTTVKLIGTWASPFAIRAQ

VALHLKSVEHEYVEETDVLKGKSDLLIKSNPIHKKVPVLIHGDVSICESLNIV

QYVDESWPSDLSILPTLPSERAFARFWAHFVDGKLFESIDAVAGAKDDAAR

MTLAGNLMENLAALEEAFQKSSKGGDFFGGGNIGFVDITVGAIVGPISVIE

AFSGVKFLRPDTTPGLIQWAEKFRAHEAVKPYMPTVAEFIEFAKKKFSV

>AtGSTU13 XP_020869675.1

MAQNETVKLIGSWSSPYSLRARVALHLKSVKYEYLDEPDVLKEKSELLLKS

NPIHKKVPVLLHGDLSICESLNVVQYVDEAWPSVPLILPSDAYDRASARFW

AQYIDDKCFAAVDAVVGAKDDEGKMAAAGKLMECLAILEETFQKSSKGL

GFFGGETIGYLDIACSALLGPISVIEAFSGVKFLRQETTPGLIQWAERFRAHE

AVKPYMPTVEEVVAFAKQKFNVQ

>AtGSTU14 NP_174034.1

MAQNDTVKLIGCSDDPFSIRPRVALHLKSIKYEYLEEPDDDLGEKSQLLLKS

NPIHKKTPVLIHGDLAICESLNIVQYLDEAWPSDPSILPSNAYDRASARFWA

QYIDDKCFEAANALTGANNDEERIAATGKLTECLAILEETFQKSSKGLGFFG

GETIGYLDIACAALLGPISVIEMFSADKFVREETTPGLIQWAVRFRAHEAVRP

YMPTVEEVTELVKQRIEEGFKRNFKSNVSTSEYE

>AtGSTU15 NP_176176.1

MGEREEVKLLGTWYSPVVIRAKIALRLKSVDYDYVEEDLFGSKSELLLKSNP

IFKKVPVLIHNTKPVCVSLNIVEYIDETWNSSGSSILPSHPYDRALARFWSVF

VDDKWLPTLMAAVVAKSEEAKAKGMEEVEEGLLQLEAAFIALSKGKSFFG

GETIGFIDICLGSFLVLLKAREKLKNEKILDELKTPSLYRWANQFLSNEMVK

NVVPDIDKVAKFIEEFEDRAQYIRCF

>AtGSTU16 NP_176178.1

MGEKEEVKLLGVWYSPYAIRPKIALRLKSVDYDYVEENLFGSKSELLLKSNP

VHKKVPVLLHNNKPIVESLNIVEYIDETWNSSAPSILPSHPYDRALARFWSD

FVDNKWFPALRMAAITKSEDAKAKAMEEVEEGLLQLEDAFVSISKGKPFF

GGEAIGFMDICFGSFVVLLKAREKFKAEKLLDESKTPSLCKWADRFLSDETV

KNVAPEIEKVAEFLQELEVRAQSAASRS

>AtGSTU17 NP_172508.4

MASSDVKLIGAWASPFVMRPRIALNLKSVPYEFLQETFGSKSELLLKSNPVH

KKIPVLLHADKPVSESNIIVEYIDDTWSSSGPSILPSDPYDRAMARFWAAYID

EKWFVALRGFLKAGGEEEKKAVIAQLEEGNAFLEKAFIDCSKGKPFFNGD

NIGYLDIALGCFLAWLRVTELAVSYKILDEAKTPSLSKWAENFCNDPAVKPV

MPETAKLAEFAKKIFPKPQA

>AtGSTU18 XP_002892562.1

MATEDVKLIGSWASVFVMRAKIALHLKSISYEFLQETFGSKSELLLKSNPVH

KKMPVLIHADKPVCESNIIVQYIDEAWNSSGPSILPSHPYDRAIARFWAAYI

DDQWFISLRSILTAQGEEEKKASIAQVEERTELLEKAFNDCSKGKPFFNGDH

IGYLDIALGSFLGWWRVVELDANHKFLDETKTPSLAKWAERFCDDPAVKPI

MPEITKLAEFARKLFPKPQA

>AtGSTU19 NP_565178.1

MANEVILLDFWPSMFGMRTRIALREKGVEFEYREEDLRNKSPLLLQMNPIH

KKIPVLIHNGKPVNESIIQVQYIDEVWSHKNPILPSDPYLRAQARFWADFID

KKLYDAQRKVWATKGEEQEAGKKDFIEILKTLESELGDKPYFSGDDFGYVD

IALIGFYTWFPAYEKFANFSIESEVPKLIAWVKKCLQRESVAKSLPDPEKVTEF

VSELRKKFVPE

>AtGSTU20 NP_177958.1

MANLPILLDYWPSMFGMRARVALREKGVEFEYREEDFSNKSPLLLQSNPIH

KKIPVLVHNGKPVCESLNVVQYVDEAWPEKNPFFPSDPYGRAQARFWADF

VDKKFTDAQFKVWGKKGEEQEAGKKEFIEAVKILESELGDKPYFGGDSFGY

VDISLITFSSWFQAYEKFGNFSIESESPKLIAWAKRCMEKESVSKSLPDSEKIVA

YAAEYRKNNL

>AtGSTU21 NP_001319402.1

MAAEVILLGFWPSMFGMRTMIALEEKGVKYEYREEDVINNKSPLLLEMNPI

HKTIPVLIHNGKPVLESLIQIQYIDEVWSDNNSFLPSDPYHRAQALFWADFI

DKKEQLYVCGRKTWATKGEELEAANKEFIEILKTLQCELGEKPYFGGDKFG

FVDIVLIGFYSWFPAYQKFGNFSIEPECLKLIAWGKRCMQRESVAKALPDSE

KVVGYVLQLKKLYGIE

>AtGSTU22 NP_177956.1

MADEVILLDFWPSPFGVRARIALREKGVEFEYREENLRDKSPLLLQMNPVH

KKIPVLIHNGKPVCESMNVVQYIDEVWSDKNPILPSDPYQRAQARFWVDF

VDTKLFEPADKIWQTKGEEQETAKKEYIEALKILETELGDKPYFGGDTFGFV

DIAMTGYYSWFEASEKLANFSIEPECPTLMASAKRCLQRESVVQSLHDSEKI

LAFAYKIRKIYCV

>AtGSTU23 NP_177955.1

MEEEIILLDYWASMYGMRTRIALEEKKVKYEYREEDLSNKSPLLLQMNPIH

KKIPVLIHEGKPICESIIQVQYIDELWPDTNPILPSDPYQRAQARFWADYIDK

KTYVPCKALWSESGEKQEAAKIEFIEVLKTLDSELGDKYYFGGNEFGLVDIA

FIGFYSWFRTYEEVANLSIVLEFPKLMAWAQRCLKRESVAKALPDSDKVLKS

VSDHRKIILGID

>AtGSTU24 NP_173160.1

MADEVILLDFWASMFGMRTRIALAEKRVKYDHREEDLWNKSSLLLEMNP

VHKKIPVLIHNGKPVCESLIQIEYIDETWPDNNPLLPSDPYKRAHAKFWAD

FIDKKVNVTARRIWAVKGEEQEAAKELIEILKTLESELGDKKYFGDETFGYV

DIALIGFHSWFAVYEKFGNVSIESECSKLVAWAKRCLERESVAKALPESEKVI

TFISERRKKLGLE

>AtGSTU25 NP_173161.1

MADEVILLDFWPSMFGMRTRIALEEKNVKFDYREQDLWNKSPILLEMNPV

HKKIPVLIHNGNPVCESLIQIEYIDEVWPSKTPLLPSDPYQRAQAKFWGDFI

DKKVYASARLIWGAKGEEHEAGKKEFIEILKTLESELGDKTYFGGETFGYV

DIALIGFYSWFEAYEKFGSFSIEAECPKLIAWGKRCVERESVAKSLPDSEKIIKF

VPELRKKLGIEIE

>AtGSTU26 NP_173162.1

MANDQVILLDYWPSMFGMRTKMALAEKGVKYEYKETDPWVKTPLLIEM

NPIHKKIPVLIHNGKPICESLIQLEYIDEVWSDASPILPSDPYQKSRARFWAEF

IDKKFYDPSWKVWATMGEEHAAVKKELLEHFKTLETELGDKPYYGGEVFG

YLDIALMGYYSWFKAMEKFGEFSIETEFPILTTWTKRCLERESVVKALADSD

RIIEYVYVLRKKFGAA

>AtGSTU27 NP_189966.1

MSEEEVVVLNFWPSMFGARVIMALEEKEIKFEYKEEDVFGQKTDLLLQSNP

VNKKIPVLIHNGKPVCESNIIVEYIDEVWKDDKTLRLLPSDPYQKSQCRFW

ADLIDKKVFDAGRRTWTKRGKEQEEAKQEFIEILKVLERELGDKVYFGGN

DNVSMVDLVLISYYPWFHTWETIGGFSVEDHTPKLMDWIRKCLTRPAISKS

LPDPLKIFDRVTQIIKVHEFFYGY

>AtGSTU28 NP_175772.1

MGKENSKVVVLDFWASPYAMRTKVALREKGVEFEVQEEDLWNKSELLLKS

NPVHKKVPVLIHNNTPISESLIQVQYIDETWTDAASFLPSDPQSRATARFWA

DYADKTISFEGGRKIWGNKKGEEQEKGKKEFLESLKVLEAELGDKSYFGGE

TFGYVDITLVPFYSWFYALEKCGDFSVEAECPKIVAWGKRCVERNSVAATLP

ESEKVYQQVLKLRQIFGVE

>AtGSTF1 P42769.1

MVTVKLYGMAYSTCTKRVYTTAKEIGVDVKIVPVDLMKGEHKEPAYLDNY

HPFGVIPVLEDEDGTKIYESRAISRYLVAKYGKGSSLLPSPSDPKAYGLFEQA

ASVEYSSFDPPASSLAYERVFAGMRGLKTNEELAKKYVDTLNAKMDGYERI

LSKQKYLAGNDFTLADLFHLPYGAMVAQLEPTVLDSKPHVKAWWAASLR

VIPGRLLRNSSKEFM

>AtGSTF2 NP_192161.1

MAGIKVFGHPASIATRRVLIALHEKNLDFELVHVELKDGEHKKEPFLSRNPF

GQVPAFEDGDLKLFESRAITQYIAHRYENQGTNLLQTDSKNISQYAIMAIG

MQVEDHQFDPVASKLAFEQIFKSIYGLTTDEAVVAEEEAKLAKVLDVYEAR

LKEFKYLAGETFTLTDLHHIPAIQYLLGTPTKKLFTERPRVNEWVAEITKRPA

SEKVQ

>AtGSTF3 NP_178394.1

MAGIKVFGHPASTSTRRVLIALHEKNLDFELVHVELKDGEHKKEPFLSRNP

FGQVPAFEDGDLKLFESRAITQYIAHRYENQGTNLLPADSKNIAQYAIMSIG

IQVEAHQFDPVASKLAWEQVFKFNYGLNTDQAVVAEEEAKLAKVLDVYE

ARLKEFKYLAGETFTLTDLHHIPVIQYLLGTPTKKLFTERPRVNEWVAEITK

RPASEKVL

>AtGSTF4 NP_849581.1

MDCLQMVFKLFPNWKREAEVKKLVAGYKVHGDPFSTNTRRVLAVLHEKR

LSYEPITVKLQTGEHKTEPFLSLNPFGQVPVFEDGSVKLYESRAITQYIAYVH

SSRGTQLLNLRSHETMATLTMWMEIEAHQFDPPASKLTWEQVIKPIYGLET

DQTIVKENEAILEKVLNIYEKRLEESRFLACNSFTLVDLHHLPNIQYLLGTPT

KKLFEKRSKVRKWVDEITSREAWKMACDQEKSWFNKPRN

>AtGSTF5 NP_001322019.1

MGINASHVPETCYHHCNQTFESSRQCFKWCQELARKDEYKIYGYPYSTNT

RRVLAVLHEKGLSYDPITVNLIAGDQKKPSFLAINPFGQVPVFLDGGLKLTE

SRAISEYIATVHKSRGTQLLNYKSYKTMGTQRMWMAIESFEFDPLTSTLTWE

QSIKPMYGLKTDYKVVNETEAKLEKVLDIYEERLKNSSFLASNSFTMADLY

HLPNIQYLMDTHTKRMFVNRPSVRRWVAEITARPAWKRACDVKAWYHK

KKN

>AtGSTF6 NP_171792.1

MAGIKVFGHPASTATRRVLIALHEKNVDFEFVHVELKDGEHKKEPFILRNP

FGKVPAFEDGDFKIFESRAITQYIAHEFSDKGNNLLSTGKDMAIIAMGIEIES

HEFDPVGSKLVWEQVLKPLYGMTTDKTVVEEEEAKLAKVLDVYEHRLGES

KYLASDHFTLVDLHTIPVIQYLLGTPTKKLFDERPHVSAWVADITSRPSAQK

VL

>AtGSTF7 NP_171791.1

MAGIKVFGHPASTATRRVLIALHEKNLDFEFVHIELKDGEHKKEPFIFRNPF

GKVPAFEDGDFKLFESRAITQYIAHFYSDKGNQLVSLGSKDIAGIAMGIEIES

HEFDPVGSKLVWEQVLKPLYGMTTDKTVVEEEEAKLAKVLDVYEHRLGES

KYLASDKFTLVDLHTIPVIQYLLGTPTKKLFDERPHVSAWVADITSRPSAKK

VL

>AtGSTF8 NP_850479.1

MGAIQARLPLFLSPPSIKHHTFLHSSSNSNFKIRSNKSSSSSSSSIIMASIKVH

GVPMSTATMRVLATLYEKDLQFELIPVDMRAGAHKQEAHLALNPFGQIPA

LEDGDLTLFESRAITQYLAEEYSEKGEKLISQDCKKVKATTNVWLQVEGQQ

FDPNASKLAFERVFKGMFGMTTDPAAVQELEGKLQKVLDVYEARLAKSEF

LAGDSFTLADLHHLPAIHYLLGTDSKVLFDSRPKVSEWIKKISARPAWAKVI

DLQKQ

>AtGSTF9 NP_180643.1

MVLKVYGPHFASPKRALVTLIEKGVAFETIPVDLMKGEHKQPAYLALQPFG

TVPAVVDGDYKIFESRAVMRYVAEKYRSQGPDLLGKTVEDRGQVEQWLDV

EATTYHPPLLNLTLHIMFASVMGFPSDEKLIKESEEKLAGVLDVYEAHLSKS

KYLAGDFVSLADLAHLPFTDYLVGPIGKAYMIKDRKHVSAWWDDISSRPA

WKETVAKYSFPA

>AtGSTF10 NP_180644.1

MVLTIYAPLFASSKRAVVTLVEKGVSFETVNVDLMKGEQRQPEYLAIQPFGK

IPVLVDGDYKIFESRAIMRYIAEKYRSQGPDLLGKTIEERGQVEQWLDVEAT

SYHPPLLALTLNIVFAPLMGFPADEKVIKESEEKLAEVLDVYEAQLSKNEYL

AGDFVSLADLAHLPFTEYLVGPIGKAHLIKDRKHVSAWWDKISSRAAWKE

VSAKYSLPV

>AtGSTF11 NP_186969.1

MVVKVYGQIKAANPQRVLLCFLEKDIEFEVIHVDLDKLEQKKPQHLLRQPF

GQVPAIEDGYLKLFESRAIARYYATKYADQGTDLLGKTLEGRAIVDQWVEV

ENNYFYAVALPLVMNVVFKPKSGKPCDVALVEELKVKFDKVLDVYENRLA

TNRYLGGDEFTLADLSHMPGMRYIMNETSLSGLVTSRENLNRWWNEISAR

PAWKKLMELAAY

>AtGSTF12 NP_197224.1

MVVKLYGQVTAACPQRVLLCFLEKGIEFEIIHIDLDTFEQKKPEHLLRQPFG

QVPAIEDGDFKLFESRAIARYYATKFADQGTNLLGKSLEHRAIVDQWADVE

TYYFNVLAQPLVINLIIKPRLGEKCDVVLVEDLKVKLGVVLDIYNNRLSSNR

FLAGEEFTMADLTHMPAMGYLMSITDINQMVKARGSFNRWWEEISDRPS

WKKLMVLAGH

>AtGSTF13 NP_191835.1

MAMKLYGDEMSACVARVLLCLHEKNTEFELVPVNLFACHHKLPSFLSMNP

FGKVPALQDDDLTLFESRAITAYIAEKHRDKGTDLTRHEDPKEAAIVKLWSE

VEAHHFNPAISAVIHQLIVVPLQGESPNAAIVEENLENLGKILDVYEERLGK

TKYLAGDTYTLADLHHVPYTYYFMKTIHAGLINDRPNVKAWWEDLCSRP

AFLKVSPGLTVAPTTN

>AtGSTF14 NP_175408.1

MADSKMKLHCGFIWGNSAALFCINEKGLDFELVFVDWLAGEAKTKTFLST

LNPFGEVPVLEDGDLKLFEPKAITRYLAEQYKDVGTNLLPDDPKKRAIMSM

WMEVDSNQFLPIASTLIKELIINPYQGLATDDTAVQENKEKLSEVLNIYETRL

GESPYLAGESFSLADLHHLAPIDYLLNTDEEELKNLIYSRPNVAAWVEKMK

MRPAWLKTVVMKNHIVDLMKQRRLPIKLDSSCHESTVVAQKNAIAIENK

>AtGSTZ1 NP_178344.1

MANSGEEKLKLYSYWRSSCAHRVRIALALKGLDYEYIPVNLLKGDQFDSDF

KKINPMGTVPALVDGDVVINDSFAIIMYLDEKYPEPPLLPRDLHKRAVNYQ

AMSIVLSGIQPHQNLAVIRYIEEKINVEEKTAWVNNAITKGFTALEKLLVNC

AGKHATGDEIYLADLFLAPQIHGAINRFQINMEPYPTLAKCYESYNELPAF

QNALPEKQPDAPSSTI

>AtGSTZ2 NP_178343.1

MSYVTDFYQAKLKLYSYWRSSCAHRVRIALTLKGLDYEYIPVNLLKGDQSD

SDFKKINPMGTVPALVDGDVVINDSFAIIMYLDDKYPEPPLLPSDYHKRAV

NYQATSIVMSGIQPHQNMALFRYLEDKINAEEKTAWITNAITKGFTALEKL

LVSCAGKYATGDEVYLADLFLAPQIHAAFNRFHINMEPFPTLARFYESYNE

LPAFQNAVPEKQPDTPSTI

>AtGSTT1 NP_198937.1

MMKLKVYADRMSQPSRAVIIFCKVNGIQFDEVLISLAKRQQLSPEFKDINPL

GKVPAIVDGRLKLFESHAILIYLSSAFPSVADHWYPNDLSKRAKIHSVLDW

HHTNLRRGAAGYVLNSVLGPALGLPLNPKAAAEAEQLLTKSLSTLETFWL

KGNAKFLLGSNQPSIADLSLVCELMQLQVLDDKDRLRLLSTHKKVEQWIE

NTKKATMPHFDETHEILFKVKEGFQKRREMGTLSKPGLQSKI

>AtGSTT2 NP_198940.3

MKLKVYADRMSQPSRAVLIFCKVNEIQFDEILISLGKRQQLSPEFKEINPMG

KVPAIVDGRLKLFESHAILIYLSSAYASVVDHWYPNDLSKRAKIHSVLDWH

HTNLRPGASGYVLNSVLAPALGLPLNPKAAAEAENILTNSLSTLETFWLKG

SAKFLLGGKQPSIADLSLVCELMQLQVLDDKDRLRLLSPHKKVEQWIESTR

KATMPHSDEVHEVLFRAKDRFQKQREMATASKPGPQSKIIQFSSIGGTSDGP

NLVQDTTDRKARRRKWSPPDDVILISAWLNTSKDRKVVVYDEQQAHTFW

KRIGAHVSNSASLANLPKREWNHCRQRWRKINDYVCKFVGCYDQALNQ

RASGQSEDDVFQVAYQLYYNNYMSNFKLEHAWRELRHNKKWCSTYTSEN

SKGGGSSKRTKLNGGGVYSSSCNPESVPIALDGEEQVMDRPLGVKSSKQKE

KKVATKTMLEEREADSRSRLENLWVLDEEEQVMDLPLGVKSSKQKERKVA

TKTMIEEREAANFRSRLGNLWLLKEKEEREADSRSRLENLWALKEKDIEEQ

KKLTRMEVLKSLLGRRTGETSEKEETLKNKLIDEML

>AtGSTT3 NP_198938.1

MKLKVYADRMSQPSRAVLIFCKVNEIQFDEILIYLANRQQLSPEFKDINPMG

KVPAIVDGKLKLSESHAILIYLSSAYPSVVDHWYPTDLSKRARIHSVLDWHH

TNLRPGAAGYVLNSVLGPALGLPLNPKAAAEAEQLLTKSLTTLDTFWLKG

NAMFLLGSNQPSIADLSLVCELTQLQVLDDKDRLRLLSPHKNVEQWIENTR

KATMPHFDEVHEVLFRAKDRCQKQREMATASKPGPQSKIIQFSTIGEKSDD

PNLVQNTTDRRKHRRKWSRAEDAILISAWLNTSKDPIVDNEHKACAFWK

RIGAYFNNSASLANLPKREPSHCKQRWSKLNDKVCKFVGCYDQALNQRSS

GQSEDDVFQVAYQVYTNNYKSNFTLEHAWRELRHSKKWCSLYPFENSKGG

GSSKRTKLNNGDRVYSSSSNPESVPIALDEEEQVMDLPLGVKSSKQKEKKV

ATIITIEEREADSGSRLENLWVLDEEEQVMDRPLGVKSLEQKENKVAPKPTI

EEREAADSRSRLENLWALKEKEEREADSRSRLENLWALKEKDIEEQKKLTR

MEVLKSLLGRTTDQLSEKEDILKNKLIDEML

>AtGSTL1 CAB86032.1

MALSPPKIFVEDRQVPLDATSDPPALFDGTTRLYISYTCPFAQRVWITRNLK

GLQDEIKLVPIDLPNRPAWLKEKVNPANKVPALEHNGKITGESLDLIKYVD

SNFDGPSLYPEDSAKREFGEELLKYVDETFVKTVFGSFKGDPVKETASAFDH

VENALKKFDDGPFFLGELSLVDIAYIPFIERFQVFLDEVFKYEIIIGRPNLAAW

IEQMNKMVAYTQTKTDSEYVVNYFKRFM

>AtGSTL2 NP_191064.1

MSVGLKVSAFLHPTLALSSRDVSLSSSSSSLYLDRKILRPGSGRRWCKSRRTE

PILAVVESSRVPELDSSSEPVQVFDGSTRLYISYTCPFAQRAWIARNYKGLQN

KIELVPIDLKNRPAWYKEKVYSANKVPALEHNNRVLGESLDLIKYIDTNFEG

PSLTPDGLEKQVVADELLSYTDSFSKAVRSTLNGTDTNAADVAFDYIEQALS

KFNEGPFFLGQFSLVDVAYAPFIERFRLILSDVMNVDITSGRPNLALWIQEM

NKIEAYTETRQDPQELVERYKRRVQAEARL

>AtGSTL3 NP_195899.1

MAPSFIFVEDRPAPLDATSDPPSLFDGTTRLYTSYVCPFAQRVWITRNFKGL

QEKIKLVPLDLGNRPAWYKEKVYPENKVPALEHNGKIIGESLDLIKYLDNTF

EGPSLYPEDHAKREFGDELLKYTDTFVKTMYVSLKGDPSKETAPVLDYLEN

ALYKFDDGPFFLGQLSLVDIAYIPFIERFQTVLNELFKCDITAERPKLSAWIEEI

NKSDGYAQTKMDPKEIVEVFKKKFM

>AtDHAR1 NP_173387.1

MALEICVKAAVGAPDHLGDCPFSQRALLTLEEKSLTYKIHLINLSDKPQWF

LDISPQGKVPVLKIDDKWVTDSDVIVGILEEKYPDPPLKTPAEFASVGSNIFG

TFGTFLKSKDSNDGSEHALLVELEALENHLKSHDGPFIAGERVSAVDLSLAP

KLYHLQVALGHFKSWSVPESFPHVHNYMKTLFSLDSFEKTKTEEKYVISGW

APKVNP

>AtDHAR2 NP_177662.1

MALDICVKVAVGAPDVLGDCPFSQRVLLTLEEKKLPYKTHLINVSDKPQWF

LDISPEGKVPVVKLDGKWVADSDVIVGLLEEKYPEPSLKTPPEFASVGSKIFG

AFVTFLKSKDANDGSEKALVDELEALENHLKTHSGPFVAGEKITAVDLSLA

PKLYHLEVALGHYKNWSVPESLTSVRNYAKALFSRESFENTKAKKEIVVAG

WESKVNA

>AtDHAR3 NP_568336.1

MISLRFQPSTTAGVLSASVSRAGFIKRCGSTKPGRVGRFVTMATAASPLEICV

KASITTPNKLGDCPFCQKVLLTMEEKNVPYDMKMVDLSNKPEWFLKISPE

GKVPVVKFDEKWVPDSDVITQALEEKYPEPPLATPPEKASVGSKIFSTFVGF

LKSKDSGDGTEQVLLDELTTFNDYIKDNGPFINGEKISAADLSLAPKLYHM

KIALGHYKNWSVPDSLPFVKSYMENVFSRESFTNTRAETEDVIAGWRPKV

MG

>AtDHAR4 Q9FG59.1

MGIEVCVKAASGAPDVLGDCPFGQRILLTLEDKKLPYKTHLIDVSLKPDWF

LAISPKGKLPLVKFDEDENWVADSDLIVGIIEEKYPEPSLVTFPPEFASVGSKII

GAFVMFLTSKDHANDGSDMALLDELEALDHHLKTHVGPFVAGDKVTVV

DLSLAPKLYHLETTLGHFMDWCVPESLTNVRDYMKVLFSLESFEKTKAAKE

YLIASWAPKLDV

>AtDHAR5 NP_173386.1

MGKSFLIGRRIKIYNLKKKSCYYYWKQAFDKYTNLMSCEDCKIILDIKCSVF

LDWPTFCDISPQGKVPVLKIDDKWVTDSDATVGILEEKYPDPPLKTPAEFAS

VGSNIFEALENHLKSHDGPFIAGERVSAVDLSLAPKLYHLQVALGHFKSWS

VPESFPHVHNYMKTLFSLDSFEKTKTEEKCVISGWAPKVNP

>AtTCHQD1 NP_177853.1

MQLYHHPYSIDSQRVRLALEEKGIDYTSYHVNPITGKHMDPSFFRMNPNA

KLPVFRNGSHIILDTIEIIEYLERIAEVSSGIEDATFNREVVEWMRKIREWESK

LFTLAHIPDNRRLYVSKFLRMVVIARMAESPDLASAYHRKLREAYDTEDKL

KDPGALRRSKDHLLRLLDEVETKLEGTTYLAGNEFSMADVMLIPVLARLSL

LDLEEEYISSRKNLAEYWALVRRRPSYKKVIGRYFNGWRKYATLVKTWMFV

RVRSLLRKY

>AtEF1Bγ1 NP_563848.1

MALVLHTYKGNKSAEKALIAAEYVGVQIDVPSDFQMGVTNKTPAFLKMN

PIGKVPVLETPEGSVFESNAIARYVSRLNGDNSLNGSSLIEYAQIEQWIDFSSL

EIYASILRWFGPRMGFMPYSAPAEEGAISTLKRALDALNTHLTSNTYLVGHS

ITLADIITVCNLNLGFATVMTKKFTSEFPHVERYFWTVVNQPNFTKVLGDV

KQTEAVPPIASKKAAQPAKPKEEPKKKEAPVAEAPKLAEEEEAPKPKAKNP

LDLLPPSPMVLDDWKRLYSNTKSNFREVAIKGFWDMYDPEGYSLWFCDYK

YNDENMVSFVTLNKVGGFLQRMDLARKYSFGKMLICGSEGPFKVKGLWL

FRGPEIPKFIMDEVYDMELYEWTKVDISDEAQKERVSQMIEDAEPFEGEALL

DAKCFK

>AtEF1Bγ2 NP_176084.1

MALVMHTYKGNKGANKALIAAEYAGVKIEESADFQMGVTNKSPEFLKM

NPIGKVPVLETPEGPIFESNAIARYVSRKNGDNSLNGSSLIEYAHIEQWIDFS

SLEIDANMLKWFAPRMGYAPFSAPAEEAAISALKRGLEALNTHLASNTFLV

GHSVTLADIVTICNLNLGFATVMTKKFTSAFPHVERYFWTMVNQPEFKKV

LGDAKQTEAVPPVPTKKAPQPAKPKEEPKKAAPVAEAPKPAEEEEAPKPK

AKNPLDLLPPSPMVLDDWKRLYSNTKSNFREVAIKGFWDMYDPEGYSLWF

CDYKYNDENMVSFVTLNKVGGFLQRMDLARKYSFGKMLICGSEGPFKVK

GLWLFRGPEIPKFIMDEVYDMELYEWTKVDISDEAQKERVSQMIEDAEPFE

GEALLDAKCFK

>AtGHR1 NP_199315.1

MARSGVDETSESGAFVRTASTFRNFVSQDPDSQFPAESGRYHLYISYACPWA

CRCLSYLKIKGLDEAITFSSVHAIWGRTKETDDHRGWVFPDSDTELPGAEP

DYLNGAKSVRELYEIASPNYEGKYTVPVLWDKKLKTVVNNESSEIIRMFNT

EFNGIAKTPSLDLYPSHLRDVINETNGWVFNGINNGVYKCGFARKQEPYN

EAVNQLYEAVDRCEEVLGKQRYICGNTFTEADIRLFVTLIRFDEVYAVHFKC

NKRLLREYPNIFNYIKDIYQIHGMSSTVNMEHIKQHYYGSHPTINPFGIIPH

GPNIDYSSPHDRDRFSS

>AtGHR2 NP_199312.1

MATPMENENPNFARTATSFRNFVSKDPDSQFPAESGRYHLYISYACPWASR

CLAILKLKGLDKAISFSSVQPLWRNTKENDEHMGWVFPDSDTEVLGAERD

HINGAKSVRELYDIASSNYTGKYTVPVLWDKKLKTIVNNESSEILRMFNTEF

NHVAENPSLDLYPPNLRAIIDETNEWIHDGINNGVYKCGFATNQETYDVE

VKRLYEALDRCEDILRKQRFLCGNTLTESDIRLFVTVIRFDEAYAVIFKCDKR

LVREYYHLFNYTKDIYQIAGMSSTVKMDHIKQNYYGSFPSINPLEIIAHGPNI

DYSLPHDRHRFSLESDYTRLELFESASFVCELKLIEIFDSL

>AtGHR3 NP_193723.3

MSYSTIISNTSFLSLASKFTTRGSRLQCTVSMARSAVDETSDSGAFQRTASTFR

NFVSKDSNSQFPAESGRYHLYISYACPWASRCLSYLKIKGLDDAISFSSVKPI

WGRTKETDEHMGWVFPGSDTEVPGADPDHLNGAKSVRELYEIASPNYTG

KYTVPVLWDKKLKTVVNNESAEIIRMFNTEFNHIAGNPDLDLYPSHLQAKI

DETNEWIYNGINNGVYRCGFAKKQGPYEEAVEQVYEALDRCEEILGKHRYI

CGNTLTETDIRLFVTLIRFDEVYAVHFKCNKKLIREYPNLFNYTKDIFQIPGM

SSTVNMNHIKQHYYGSHPSINPFGIIPHGPNIDYTSPHDRHRFSK

>AtGHR4 NP_001031671.1

MSYSTIISNTSFLSLASKFTTRGSRLQCTVSMARSAVDETSDSGAFQRTASTFR

NFVSKDSNSQFPAESGRYHLYISYACPWASRCLSYLKIKGLDDAISFSSVKPI

WGRTKETDEHMGWVFPGSDTEVPGADPDHLNGAKSVRELYEIASPNYTG

KYTVPVLWDKKLKTVVNNESAEIIRMFNTEFNHIAGNPDLDLYPSHLQAKI

DETNEWIYNGINNGVYRCGFAKKQGPYEEAVEQVYEALDRCEEILGKHRYI

CGNTLTETDIRLFVTLIRFDEVSSYFQSKKKKYTICERISNVETLIQVYAVHFK

CNKKLIREYPNLFNYTKDIFQIPGMSSTVNMNHIKQHYYGSHPSINPFGIIP

HGPNIDYTSPHDRHRFSK

>AtGSTM1 NP_565446.1

MEGDQETNVYTLVARKPSFDLPTACPNCLPAYIYLKLAQLPFELAFNSTFPD

SDELPYFESDTYVAYNNEDGGVIEKLKKDGIVNLDSQLQSLSDYLSLKALIVS

WLEEALTYEIWVGTEGISTSKIYYSDLPWVISKVLFYKQTYLAKNRLGITKEN

AEQREKQIYKRASEAYEALSTRLGEQKFLFEDRPSSLDAFLLSHILFIIQALPV

TSVLRCKLLEHSNLVRYAEKLKSEFLEASSSSPSPPLHSFPSSFPRKSSKPKSKP

KVEKTEEEKKFKKRARFFLAAQFLAVVIYVSVMGGGSSDELEYEDEDD

> SlGSTU1 Solyc01g081250.2.1

MADEVKIYRNWSSPYGLRVVWALDIKGIEYENIFEDLSQKSPQLLQYNPVH

KKIPVLVHKGKPICESLVILEYIDETWKETTPLLPKDPYEKAIARFWAKFVDD

KLLPSIWSVFTEKRYEAKKEALVPAMQNLEFIEEQLKGKKFFGGESIGYVDL

ALGWMAYLLDVFEEVLDLKLFDADKFPLLSGWTKNFCDAPAIKQHLPPRD

KLVTKFQLFHEKFQTAN

>SlGSTU2 Solyc01g081260.1.1

MADEVKLYRTWSSRFSLRIIWALHIKGIEYEAIFEDLSHKSPQLLKYNHVHK

KFPVLVHNDKPICESLVILEYIDETWKETSHLLPQDPYEKAMARFWAKFVD

DKFKNLNVLNHIIYGPKQNFIIN

>SlGSTU3 Solyc01g081270.2.1

MADEVKLYRTWSSPFGLRIVWALHIKGIEYEAIFEDLSQKSPQLLQYNPVHK

KIPVLVHKGKPICESLVILEYIDETWKETAPLLPQDPYEKAMARFWAKFVED

KLLPSVWSIFTEQGYDAKKEAFVPAVQNLEIIEEQLKEKKFFGGESIGYVDLV

LGWMAYLLDVFEEVLDLKLFDADKFPLLSGWMKNFCDAPAIKQHLPPRD

KLVTKFQLLHEKFQTAN

>SlGSTU4 Solyc01g081310.2.1

MASEKVKLLGYWASPFALKVHWALKLKGIEYEYQEEDLSNKSPLLLQYNP

VHKKIPVLVHNGKPIAESLVILEYIEETWKHNPLLPEDPYERAKARFWAKFV

DDKCVPGIFGTFAKVGVEQQKIAKEARENLKILEDELGKKHFFGDAKIGFM

DVTSAWIICWAQIVEEVVDIRLIDAEEMPSLVSWFQNVLEAAPILKECTPPK

DKLLEHNKGFHEMLVASAPS

>SlGSTU5 Solyc01g086680.2.1

MGEVKVHGIFAGPFNKRVELALKLKGVKYEYIEEDRSNKSDELVKYNPIYK

QVPVLVHNGKPICESIIILEYIDDTWENNTIPLLPKHPYQRSMARFLAKLIDE

KLMGAMYKVCYGKGEEREKGCDETFEVLKYLDNELQNKKFFGGDSIGFV

DIVASYIALWFGAIQEAIGMELLTEQKFPKLSKWIDEFLCCRIVKENLPNREV

LVPLYKAQFAAATQKASS

>SlGSTU6 Solyc01g086680.2.1

MEDVKLLGTKESIFTQRIIWALKLKGICYEFIEQDFSSRSSPLLVKLNPVYNKV

PVIVHDGKSLAESLVILEYIEETWPLINPLFPLDPFQRASTRFWARFVDGKFY

EAAKKAFFSSGETKAEGVESVVEGLHLLEGQIIGKKFFGGEKIGYLDIITGWI

AYWFQYIEEIGEFKAMDSTKYPCLHAWINNFIQLPIIKQSLPTPDVVKSVFRG

FKDAAALADAN

>SlGSTU7 Solyc02g081240.1.1

MSKDDELRLLDFWASPFCMRVKIALSEKGVAYESQQEDLFGGKSDMLLKS

NPIYEKVPVLLDNGKPIVESNNIVYYIEDKYPSTNPLLPSCAYGRSRARFWA

DFIDKKIFEGGMCIWKSKGEELEIAKKDFIEILKKLEGAMGDKDFFGGDNF

GYVDVIAIAMTSWFHAYEVFGDFKVEQECPKFGCWMKRCLERESVSSVLP

DPEKIYQCVVMLRKMHGIE

>SlGSTU8 Solyc03g116120.1.1

MGEENKVTLHGMWLSPYVKRVELALKVKGIPFEYIEEDLSNKSPLILKYNPI

HKKVPILVHNGKPVNESFVIVEYIDETWKNGPQLLPEDPYERSKVHFWAAY

IQQVMESMLNIFTAEDQKQACNEFHQKFRLLEDGMKNFFPTIENRNIGLID

IWIVVAFGMCKAQEEAFGVNFLDPEKVPLIHSRVNSLLELPLLRETVPDHD

KAVSFLRALKETSTKGQAH

>SlGSTU9 Solyc03g116130.1.1

MEEENDRVTLHGMWISTYAKKVELALKIKGIAFDYVEEDLSNKSSLLLKYN

PIHKKVPLLLHRGKPLSESLVILEYIDETWNNLQPLLLPEDPYERATVRLWAS

YCLQISDTMKKAFISARDVEGGAFDELFEILKVMEEGMKDFFPGGRSKICAE

NLGLLDIIIVCSLATYKAAEEVVGTKILDPEKNPFVYSWVTTLLELPLVKETL

PPHDKLVSRLDFIKKNGFRFQSNI

>SlGSTU10 Solyc05g006730.2.1

MAKNNLKILGAWPSPYVMRPRIALNIKCLAYDFLEEQFGTKSELLLKSNPIY

KKIPVLIHDGKPICESLIIVQYIDENWTNFGHSILPSHPYDRAIARFWAFYIDD

KWFPALCGVAAAQDEDAKKAAMETVIEGLVLLEDVFKINSKGKKFFGGDK

IGYLDIALGCFLGWLKVNEKLNNVNLLDESRTPSLYQWAKDFCVDSVVKD

VMPETDQLVQAAKIIWAKI

>SlGSTU11 Solyc05g006740.2.1

MRPRIALNVKSVCYDFLEEQLSSKSDLLLISNPVYKKIPVLIHDGKSICESLNI

VQYIDEKWTNSGPSILPLDPYDRAIARFWACYIDDKWFPLFRSFAVAQGED

AIKTALEPVFDGLVLLEDAFKNCSKGKKFFGGDKIGYVDIALGCFLGWMRV

IEKMNNVTLLDEAKTPGLYNWAEDFCADSSVKDVMPETNKLAEAAKNLIP

KIRANASS

>SlGSTU12 Solyc05g006750.2.1

MATNSVKLLGTWACPYVYRVEIALKMKSIEYEFIQERVFNKSELLLKSNPVY

KKIPVLFHDEKPICESLVILQYIDEAWLNGPAILPSDPYDRAIARFWAAYIDE

KWYPLVADYRNVEGKEAKAAMVEKISEGTLLLEEAFINMSKGKSYFGGDSI

GYVDIVFGSLLGWVKVIEIVDELKILDETKTPSLAEWDEKFCSHNVVKDIIPE

TEKLVEIYHKYVELKKANLS

>SlGSTU13 Solyc05g026210.1.1

MATSIIKVLGTPASPFANRVSIALNVKSVDYEFVQEDMSNKSELLLKSNPVY

KKIPVLILGENIICESLVIVQYIDETWTNGPSVLPSNPLDRAITRFWVAYIDCK

WLPLMSDLGKAQGEEAILEVQEKLQQALVPLEEAFVKCSKGKSFFGGENIG

YIDIALWCILGWIKAIKIMLGIEIFNVTKAPELVNWGNRFLEDKCVKGAML

EPEKLVEIVKLHLAKKEANNAN

>SlGSTU14 Solyc05g026220.1.1

MNIFKVNGWTCTPPLQLSLKIKSIEYEFIQEHILNKSELLLKSNPVNKKIPILF

HDEKPICESLVILQNIDEPWLNGPSILSSDPYNRSIARFWAAYIDDKEYRNAE

GKEAKAVVVDKMSEGNMLLEETFIKISKGKSFFSGDSIGYVDIVLGSLLGWV

R

>SlGSTU15 Solyc06g069040.2.1

MGQVKLIGSSGSLFCTRVEWALKLKGVDYEYIQEDLLNKSELLIKSNPVHK

KIPVLLHDDKPVVESLLILEYIDETWKGYPLLPQDPHERATARFWAKFVDD

KCVIGSWEAMAMQDEGEAKTKAIESIQELYAFIEKQIEGKKFFGGEQIGYLD

LVMGWKTLWLSAMEEVGNVKLLDPEKFPSLHQWAENFKQIPIINECMPQQ

ETLVNYFQVGLNYLRSLAANKP

>SlGSTU16 Solyc07g021460.1.1

MKEENNVTLHGMWAKSTNTVHNGNQICESSVIIEYIDETWKNESPLFPQD

PYQRIKVRFWASYIHQVYDCMLKVFRGKEALEGFYAKLSVLEDGINNFSLGI

TSNMNNIGMLDIMIVITLGAYRVQEEVFGFKLLEEENTSLLYSWVTTLIELPI

VKGITPPHEKVVSFLQYLKNKVFKAPPHAS

>SlGSTU17 Solyc07g049330.1.1

MTGRRVWSGKGEDQEEAKELIEIFKTLEGELGDKTYFGGDEKLGFVDVTCA

NFSIEAECPKLVAWAKRCMEIENVSNSLTHPHKIYGYVLELKHKVGLA

>SlGSTU18 Solyc07g056420.2.1

MANDEVILLDFWPSMYGMRLRIALAEKEIKYEYRDEDLRNKSPLLLQMNPI

HKKIPVLIHNGKPICESIIGVEYIDEVWKDKAPLLPSDPYERAQARFWADYI

DKKLYATGSKIYTATGDEQEAGKKDFVEILKVLEGALGEKPYYGGDNFGFG

DIALIGFYCWFHAYEVYGNFSIEAECPNLVAWAKRCMQRDSVAKTLPDQH

KIIEFVKILREKLGLE

>SlGSTU19 Solyc07g056430.2.1

MNPIHKKIPVLIHNGKSICESIIGVEYIDEVWKDKAPLLPSDPYERAQARFW

ADYIDKKLYRSARKIWGTKGEEQEAGKKDFIEVKVLEGALGEKPYFGGDNF

GFVDIALIGFYSWFHSYETYGNFSTEAECPKFVYHQF

>SlGSTU20 Solyc07g056440.2.1

MANDELILLDFWASMFGMRLRIALAEKGIKYEYKEEEGLISNKSALLLEMN

PIHKKVPVLIHNGKPICESIIGVEYIEEVWKDKAPLLPSDPYERAQARFWVD

YIDKKLYVSARKIWGTKGEEQEAGKKDFIEVLKVLEGELGEKPYFGGDNFG

FVDIALIGFYSWFYAYETYGNFSAEAECPKFVAWAKRCMQRDSVAKSLPDQ

HKVLEFIQMLRRKFGIE

>SlGSTU21 Solyc07g056450.2.1

MANDEVILLDFWPSMFGMRLRIALAEKEVKYEYKEEDVWNKSPLLLEMN

PIYKKVPVLIHNGKPICESIIGVEYIEEVWKDKAPLLPFDPYERAQARFWAD

YINKKCETYGNFSLEAECPKLVAWAKRCMQRDCGQVFA

>SlGSTU22 Solyc07g056460.2.1

MANDEVIVLGFWPSMFGLRLRIALAEKEVKFEYREEDLKNKSPLLLQMNPI

HRKIPVLIHNGKPICESIIGVEYIEEVWKDKAPLLPSDPYERTQARFWADYID

KKFYWPARKLWTTKGEEQEIAKKDFIECLKVLEGVLGDKPYFGGDNFGFV

DIALIGFYCWFSAYETYGNFSTEAEFPKFFAWAKRCMQRDSVAKSSPDQHK

VLEFVKVVRQRLGIE

>SlGSTU23 Solyc07g056470.2.1

MANNEVILLDFWPSMFGLRLRIALAEKEVKYEYREEDLPNKSPLLLQMNPI

HKKIPVLIHNGKPICESIIGVEYIDEVWKDKAPLLPSDPYERAQARFWADYI

DKKFYWASRKLWTTKGEELDAAKEEFIVCLKVLEGALGDKPYFGGDNFGF

VDIALIGFYCWFSAYETYGNFSTEAESPKFVAWAKRCMQRDSVAKSSPDQH

KVLEFVKVVRQRLGIE

>SlGSTU24 Solyc07g056480.2.1

MANDEVILLDFWPSMFGMRLRIALAEKEIKYEYRDEDLRNKSPLLLQMNPI

HKKIPVLIHNGKPICESIIGVEYIDEVWKDKAPFLPSDPYERAQARFWADYI

DKKLYDSGRKLWTTKGEEQETAKKDFIECLKVLEGALGEKPYFGGDNFGC

VDIALIGYYSWFYAYESYANISVEAECPKFVAWAKNCMLRDSVAKSLPDQH

KVCEFVKVLRQKFGIE

>SlGSTU25 Solyc07g056490.2.1

MADEVVLLGTYVSMFAVRVKIALAEKGIQYEYKEENLVNKSPLLLQMNPIH

KKIPVLIHNGKPICESLIIVEYIDEVWNDKSPLLPSDPYKRAQARFWADYVD

KKIYDGGKKIWTTKVEEQEAANKEFIECLKVLEGELGDKPYFDGESFGFVD

LALIPYYSWFPAYEKFGKFSIEPECPKFVAWANRCMQKENVSKYLSDPDKIY

DFVVMLRQRIGIA

>SlGSTU26 Solyc07g056500.2.1

MGDEVVLLDTFVSVFGMRVRIALAEKGIQYEYKEEDLMNKSQLLLQMNPI

HKKIPVLIHNGKPICESLIIVEYIDEVWKDKSTPLMPSDPYKRAHARFWADY

IGKKIYDGGMKIWSSKVEEHKTANKDFIECLKVLEGELGDKPYFDGKNFGL

VDMAFIPYYSWFPVYKKLSNLNIEAECPKFVAWAKRCMQKESVSKTLVDPD

KIYEFIVLFRQKIGVA

>SlGSTU27 Solyc07g056510.2.1

MGDEVVLLDLWVSPFGMRVRIALKEKGINYESKEENLSNKSSLLLKMNPIH

KQIPVLIHNGKPICESLIIVQYIDEVWKDKAPLLPSDPYERAHAKFWADYVD

KKIYSTGRLVWTTKGEAQEAAKKELIHHFKLLEKELGDKTFFGGDQFGLVD

IALIPFYSWFYALETCGNFSMIHECPKLVEWAKRCMERESVSTSLPDQYKVY
DFILELKKKLDLD

>SlGSTU28 Solyc08g062570.1.1

MEEENKVTLHGMWTSPYVKRVERALKVKGIHYEYVEEDLMNKSELLLTY
NPIHKKVPILVHNGNPICESSVIIEYIDETWKNESPLFPQDPYQRAKVRFWAS
YIHQVLLLYSTFLLSSQIHIKQTNPYTIFLIDLYIS

>SlGSTU29 Solyc09g011490.2.1

MVDVKLLGLWYSPASHKVEWALKLKGVKYEFIEENLQNKSPLLLESNPVH
KKIPILIHNGKPICESMIILEYIDETFEGPSILPKDPYDRALARFWAKFLDDKV
GAMVNTFLLKGEEQEKGKKEVCEMLNVLDNELKGKKFFVGDKFGYADM
AANFVGYWLGVFQEASGVVLVTSEKFSNFCVWRDEYVNCSQVKEYLPPR
NDLLAFVEARTQASASKA

>SlGSTU30 Solyc09g011500.2.1

MADVKLLGLWYSFFSHRVEWALKIKGVKYEIIEEDLQNKSPLLLQSNPIHK
KIPVLIHNGKSICESMIILEYIDETFEGPYILPKDPYDRALARFWAKFLDDKV
GAVVSTFIRKGEEREKGKEEACEMLKVLDNELKDKKFFVGDKFGFADIAA
NLVGYWLGIFQEASGVELVTSEKYPNFCAWRDEYMNCSQVKEYLPPRNDE
LLAFFQGCAAAASASTQN

>SlGSTU31 Solyc09g011510.2.1

MVILEYIDETFEGPSILPKDPYDRALARFWAKFLDDKVVTVVNAFLGKGEE
NEKAKEEVYEMLKILDNELKNKKFFVGDKFGIADIVANLVGLWLGVFQEG
SGVELVTSEKFPNFCSWRDEYVNCSQVKEYLPRRDDLLAFFQAFTRAQAA

ASASTQK

>SlGSTU32 Solyc09g011520.2.1

MAQVKLLGFWYSPFTHRVEWALKIKGVKYEYIEEDRYNKSPLLLESNPIYK

KVPVLIHNGKPICDSIVILEYIDEIFEGPSILPKDPHERALARFWAKFLDDKV

GAVVNTFLRKGEEQEKGKKEVCEMLKVLDNELKDKKLFVGDKLGFADMV

ANLVGLWMSVFEEASEVVLATNENFPNFCAWRNTYISCNQVKEYLPLRIDE

LLAFYQDRVRALATTLATPQK

>SlGSTU33 Solyc09g011530.1.1

MLKVLDNDFKDKKLFVGDKFGFVDIVANLVELWMGVFQEATGVVLATNE

NFPNFCARRDTYMNCSQVKEYLPSRIDELLVFYQAYIRHSSYNFCFS

>SlGSTU34 Solyc09g011540.2.1

MVDEVKLLGVSGSSYSRRVEWALRVKGVKYEFIEEDLQNKSPLLLESNPVL

KKIPVLIHNGKSICESMVIVEYIDETFEGPSILPKDPYDRAIARFWATFLDGM

CLDAVRKGLWSKREEKEKNIQEEAYEMLKIVDNELKDKKFFSGDKIGFVDV

AANYIPFWVEIVEEATGNVLITSEKFPNLCAWIDKYLKCSEVQENLPDRDM

MLSFFKAKALAEIGAK

>SlGSTU35 Solyc09g011550.2.1

MADVKLLGLWYSPYSHRVEWALKIKGVEYEFIEEDLRNKSPLLLESNPIYKK

IPVLIHNGKPICESMVIVEYIDETFEGPSILPKDPYDRAIARFWAKFFDEKGSS

VGRSFFLKGEEQEKAKEELHEMLKVVDNELKDKKYFVVDKFGFVDIVANV

VALWLGVLEEASGVVLVTNEKYPNFYAWRDEYINCSENKKYLPSRNELLAK

FKARILAASVAE

>SlGSTU36 Solyc09g011560.2.1

MEEVKLLGLWYSPFCHRVEWALKVKGVKFEFIEENLQNKSPLLLESNPIHK

KIPVLIHNGKSICESMVIVEYIDETFEGPSILPKDPYDRVIARFWVKFFEDKGS

AVGTSFFHKSEKAKEEVCEMLKILDNELKDKKFFVGDKFGFADIAANFLAL

WMGILEEATGIILVTKEKYPNFYAWRDEYINGNKEYLPSRDELLAFFKARFQ

AAATPPYSN

>SlGSTU37 Solyc09g011570.2.1

MGDVKLLGLWYSPFSHRVEWALKFKGVQYEFIEQDLQNKSPILLESNPIYK

KVPVLIHNGKPICESIVILEYIDEVFEGPSILPKDPYNRALARFWVKFFEDKG

PSMRKSILLKGEEQEKAKEEVFEMLRILDNELKGKKFFVGDKFGFVDIVAN

AGALWLGVLEEVSGVVLVTKEKFPNFCVWRDEYCTQNKEYLPSRDELLIRF

KTYI

>SlGSTU38 Solyc09g011580.2.1

MADIKLLGLWYSPFSKRVEWALKTKGVEYEYIEDDLQNKSLLLLQSNPIHK

KVPVLIHNGKPICESSVILEYIDETFEGPSILPKDPYDRALARFWAKFFEDKW

PSMMKSLFFKGEEQEKGKEEVNEMLKILDNELKDKKFFVGNNFGFVDVVA

NAVALWFGVLEEVIGVVSVTSEKFPNFCDWRDEYYIQNKEYLPSRDELFAH

YQAYIQRVAASK

>SlGSTU39 Solyc09g011590.2.1

MAEVKLLGLSYSPFNHRVEWALKIKGVKYEFIEEDLQNKSSLLLESNPIHKK

IPVLIHNGKPICESMVILEYIDEAFEGPSILPKDPYDRALARFWAKYVDDKGS

AVWKSFFFKGEEQEKAKEEAYEMLKILDNEFKDKKYFVGDKFGFADIVAN

GAALYLGILEEVSGVVLATSEKFPNFCAWRDEYCIQNKEYFPSRDELLIRYRA

YIQPVDASK

>SlGSTU40 Solyc09g011600.2.1

MADVKLIGLWYSPFSRRVEWALKIKGVEYEYIEDDLHNKSLLLLQSNPIHKA

VPVLIHNGKPLCESSVILEYIDETFEGPSILPKEPYDRSLARFWAKFFDDKGL

AIRKSIFFKGEEQEKAKEEVYDMLKVLDNELKNKKIFVGEKFGFVDIVANA

AALWLGVLEEASGVVLVTREKYPNFCDWRDEYCTQNKKYLPPRDELLAH

YQVYIQRVTTSK

>SlGSTU41 Solyc09g011610.2.1

MVIVEYIDKTFEGPSIIPKDPYDCAIARFWAKFLDDKMPPVGKSFFLKGEEQ

ERAKEEAYEILKILDNELKGQEVLCW

>SlGSTU42 Solyc09g011620.1.1

MGDVKLLGLWYSPFSHRVEWALKIKGVQYEFIEQDLQNKSPLLLESNPIHK

KIPVLIHNGKSICESMVIVEYIDETFEGPSILPEDPYDRALARFWVKFLEDQIA

AVGKSIFLKGEEQEREKKAACEMLKILENELKDKKFFVGDKFGLADIAANV

LAIWLGVFEEASGVALVTSENYPNLYGWRNEYCNQNKEYLPSRDELLIHFQ

PRFPAKAK

>SlGSTU43 Solyc09g011630.2.1

MAGVKLLGISLSPFSRRVEWALKIKGVEYEFVEEDLHNKSPVLLELNPIHKK

IPVLIHNGKPICESMVIVEYIDETFEGPSILPKDPYDRAIARFWAKFFDDKCM

PVMGKAIFGSGEESNKAKEELGDLIKILENELKDKNFFVGDKFGFADMAG

NLMAYWMGIVEEASGNIFVTSEKFPIFCNWRNEYVNCSTIKEYLPPRDEILA

HFKARFAAAQK

>SlGSTU44 Solyc09g011640.2.1

MGKIKLLGVSLSPFTHRVEWALKIKGVEYELIVEDPQNKSPLLLEYNPIHKKI

PVLIHNGKPICESMVIVEYIDETFEGPSILPKDPYDRATARFWAKFLDDKCLP

TMGKALLGNEEEKEKAKEECGELLKILDNELKDKEFFVGDKIGFVDIAAN

ALAFWMGIIEEASGVILVKNEKFPNYYTWRDNYINCSQVKKYLPSRDELFS

HFQSRFHSASTTK

>SlGSTU45 Solyc09g011650.2.1

MLGMKQEVYTQGIEYEFIEAQRPIKKCPNIIKYNPIYKKVPVFLHKGNPIPES

LVILEYIDENWKDGTSLLPKDPYQRAIARFWAKFIDEKCLPEILKLCYDSNYE

VKVKAMGELQELLKLLENELMKDNNKIFFGGENKVGYMEIVSILITYWLG

VMQEALGVDILNKKEFPNICGWADKVISFSFMKENLPPREKLLAIYKEYAQ

PLVPPNNEINHTHK

>SlGSTU46 Solyc09g063150.2.1

MEEQVKLFGAFPSPFSHRIIWALKHKNISYEYIEEDLSNKSQHLLTYNPIYKM

IPILVHNEKPIVESTIILEYIEETWPQNPLFPKDPYEKAKARFWIKFGEDKNSE

FHQIFHKIGEEQVKATENAKKILKIIEEQGLGDKKFFSGDTIGLIDIAFGWLA

FWLEVIQEAAGVKVYEPNNFPHLQSWINNFKQVAIIKENIPNRNAMLDYF

KLRRDMIVAL

>SlGSTU47 Solyc09g091130.2.1

MDQDLKLHGSWASPYSLRIIWALKLKGLLYEYIEEDLANKSDLLLKYNPIFK

KIPILVHDGKPICESMIILEYLDQIWPNQYPLLPIDSYQRALARFWVNYFEQK

SVSLWMIFRSKGEEQEKAVKDSLEMLKIIEENAFKNQKNNIFFIGGKIGIVDI

SFGWICHWLKIIEDVGGVKLIEENSFPNLQNWMKKFKEVPLIKESLPNHQK

LFLPFKLIRDMLLAS

>SlGSTU48 Solyc09g091140.2.1

MDEVKLHGTSYNLFTYRVIWALKLKGIPFEYIEEEHSNNGSLIMKYNPVFKR

FPILFHGEKVISESMVIIEYIEDTWPQNPLLPIDPLDRSIARFWVKFAGDKGA

CVGTMYYTSGEKQEKAIKETMEMLKIIEEQAFIEDEENIFFGGEKIGIVDLAF

GVIPHWLEIIEDIIGVKLLEPNLFPNLLNWVQNFKEEQIIKENLPNYEEMFVF

LKNPKKMKLSSS

>SlGSTU49 Solyc10g007620.1.1

MANEEVILLDFWPSMYGMRVRVALAEKCVNFEYKEQNMIEKSPILLEMNP

IYKKIPVLIHNGKPICESLNVVQYIDEVWKNKVIFLPSDPYEKYQAMFWAD

YVEKVFDTGRKLWMEKGGEKQTRKGNYIDTLRMLEGIIGDKLYFGGEKFG

YLDICLIGICSWFYTYEKFGEFSTEVETPKIIAWMKRCMKRESVYKNVVEPLK

VYDFALQLRKHYGIE

>SlGSTU50 Solyc10g007640.2.1

MENDEVILLDFWPSMFGMRVRVALAEKAIEYEYKEEDLFTSKSPLLVKMNP

IHKKIPVLIHNGKPVCESFVVVEYIDEVWKDKAPLLPSHPYDRSQARFWAS

YTDKLYDFGRRIWTVKREEFAEGKKDFIDPLKLLEEAALGDKPYFGGESFGF

VDIALIGFYSWFYTYETICNFSIEAECPKIAAWGKRCMKRESVSKSLADSRKI

YEVVIEFRKKNGLE

>SlGSTU51 Solyc10g084960.1.1

MSRVKLLGVYGSPASQRVEWALKIKGVKYEFITEDLQNKSPLLLKSNPVYK

KIPVLLHNDKPIAESLVIIEYIDEAFEGPSILPKDPYDRAIARFWVKFLDEKCL

PAVWKALWSQGDEQEKDKEEAYEVLKVIDNELKDKKFFGGDNIGFVDVV

ANFVGFWIGIVEEATGVVLVTSENFPNFCAWRDEYLNCDRVKENMPSREM

LLGYFKSRVQAVAAISK

>SlGSTU52 Solyc12g011300.1.1

MEEEVILLDFWCSMYGMRARIALEEKGVKYEYKEEDLKNKSPLLLQMNPI

HKKIPVLIHNGKSICESLVIIQYIDDVWKDIGPLLIPKDPYDKAQAWFWSDY

MDNTVHEYARKTWATKGEEQEQAIKDFLGGLKLLEGVLGDKPYFGGENF

GFLDVSLIGYYSWFLAYETFGKFNVELECPKLISWVKRCMERESVSKALPDS

KKVCEFVLHLRNKIGLE

>SlGSTU53 Solyc12g011310.1.1

MSNEVVLLSAYVSMFGMRVRIALHEKGIQYEYKEEDLSNKSELLLQMNPIH

KKIPVLIHNGKPICESLIIVEYIDEVWKDKSPLMPFNPYKRAQARFWADFID

KKVYDSGKRIWATKGEDQEAAKKEFIEYLKLLEGELGDKTYFNGENFGFVD

LALIPFYSWFPTFEKFGNFNIEKECPKFVAWANKCIYKDSVSKSLAESNKVY

EYVLKMKQHLGLP

>SlGSTU54 Solyc12g011320.1.1

MTQEEEVVLLDYWASPFGTMARIALVEKGVNFIHKFEDLSNKSPLLLEMNP

VHHKIPVLVHKGKSICESNIIIQYIDEIWKNNSPLLPYEPYQRAKARFLVDFI

NKKVHGSSVKVWMGQIEEQENGKKELVECSKFLEEELGDKLYFGGDVFGF

VDIALVPFYNWFIVFKTFANFNTIEIQCPKLVMWGERCLNRDSVSKSLPTSN

QVYQAYLDFKKGW

>SlGSTU55 Solyc12g062730.1.1

MEEENKVTLHGMWANPYVKRVELALKVKGIPYEYVEEYLMNKSELLLTYN

PIHKNTWKNESPLFPQDQYQRAKVRFWASYIHQVYDCMLKVFRGKEALK

RFYAKLSVLEDGINNFSLGITSNMNNIGMLDIMIVITLGAYKVQEEVFGFKL

LEEENTPLLYSWVTTLIDLPIVKGITPPHDKVVSFLQYLKNKVFKAPPHAS

>SlGSTU56 Solyc12g097080.1.1

MEIVKLIGTPFSFFTYRVIWALKLKGINYEYIDEDMSKKSSLLVKYNPIHKKV

PVLIHGDKIICESMVIVEYINETWKLNPLLSTDSYERATSRFWAKYIEEKSHSS

WNVFCYTGEKQQNAIKESLEMFKTIEENALGENNILFGGENIGFVDIAFGG

YSLWMEIIEEIVGIKLLNPHNFPRINNWIKKFKEVQTIKDNLPNRDEMFVY

MKNARGRMLASP

>SlDHAR1 Solyc05g013950.1.1

MAVLRFPSLFYLGLLLRGSKIFSTFVSFMKSKDSSDGTEQALLDELKALEEHL

KVHGPYVDGKNVCSVHMILAPKLYHLEVALGHFKKWSVPESLSHVRNYM

NDFLGSGANFPVGNVSCFLFYSRHVAAFVIAEMKIVLFI

>SlDHAR2 Solyc05g054760.2.1

MVVEVCVKAAVGAPDVLGDCPFSQRVLLTLEEKKVTYKKHLINVSDKPKW

FLEVNPEGKVPVINFGDKWIPDSDVIVGIIEEKYPNPSLIAPPEFASVGSKIFP

TFVSFLKSKDSSDSTEQALLDELKALEEHLKAHGPYINGQNVCSVDMSLAP

KLYHLEVALGHFKKWSVPESLSHVRNYMKLLFERESFQKTKAEEKYVIAGW

APKV

>SlDHAR3 Solyc06g075520.2.1

MESKDSSDCTEQALFDELKALEEHLKAHGPYVNGQNVCSVDMSLAPKLY

HLKVALGHFKKWSVTESLTHVRNYMKDFVGNMSCFLFYSRDVAASVVVE

MKIVFIYLTHWP

>SlDHAR4 Solyc09g056180.2.1

MKSKDSSDCTEHALFDELKALEEHLKAHGPYVNGQNVCSVDMSLAPKLC

HLEVALGNFKKWSVTESLSHVRNYMKNDIKSAFWSMLLCYIEQHVSHSLF

SSMQ

>SlDHAR5 Solyc11g011250.1.1

MSTAKITPSAASFATSIKHLAGIQLPRCQSTIFTSNSTKFRAPRRGFTVSMAAS

IETPLEVCVKQSITTPNKLGDCPFTQRVLLTLEEKHLPYDMKFVDLSNKPD

WFLKISPEGKVPLIKLDEKWVPDSDVISQALEEKFPKPPLTTPPEKASVGSKI

FPKFVAFLKSKDSGDGTEQALLDELTAFNDYLKENGPFINGNEVSAADLSL

GPKLYHLEIALGNYKNWSIPDSLSYMKSYMKSIFSRESFINTRALKEDVIEG

WRPKVMG

>SlDHAR6 Solyc11g039930.1.1

MKSKDSSDCTVQALFDELKALEEHLKAHGPYVNGQNVYSVDMSLAPKLY

HLEVALEHFKKWSVTECLSHVRNYMKLDPFLLPVVQGSILVIQLNT

>SlGSTF1 Solyc02g081340.2.1

MVVKVYGSAMAACPQRVMVCLIELGVDYELIHVDLDSLQQKKPDFLLLQP

FGQVPVIEEGDFRLFESRAIIRYYAAKYEDKGKKLTGTTLEEKALVDQWLEV

ESNNYNDLVYNMVLQLLVFPKMGHKSDLIVVQKCANNLEKVFDIYEQRLS

KSKYLAGDFFSLADLSHLPSLRFLMNEGGFAHLVTQRKYLHDWYLDISSRP

SWSKVLDFMNLKKLEMLPGPPKEEVKV

>SlGSTF2 Solyc06g009020.2.1

MAIKVHGPMMSPAVMRVVATLKEKDLDFELVPVNMQAGDHKKEPFISLN

PFGQVPAFEDGDLKLFESRAITQYIAHTYADKGNQLLPNDPKKMAVMSVW

MEVEAQKFDPIGSKLGFEIVIKPMLGMVTDDAVVAENEEKLGKLLDVYESR

LKESKYLGGESFTLADLHHAPSLHYLSGSKVKSLFDARPHVSAWVADILAR

PAWSKTIELSKQ

>SlGSTF3 Solyc06g009040.2.1

MAIKVHGPMLSPAVVRVVAMLKEKNLDFELVHVDLQNGDQKKEPFISLNP

FGQVPAFEDGDLKLFESRAITQYIAHTYADKGNQLLPNDPKKMAIMYVWI

EVEAQRFEPVVSKLCYEIVIKPLLDMVTDDAIVAENEEKLSKLLDVYESRLK

DSKYLGGDSFTLADLNHAPALHYLMGTKVKSLFNARPHVGAWVANILAR

PAWAKSLELTK

>SlGSTF4 Solyc09g074850.2.1

MRVISCLIEKDLDFEFVFVDMAKEEHKRHPFLSLNPFAQVPAFEDGDLKLFE

SRAITQYIAQVYASNGIQLILQDPMKMAIMSVWMEVEGQKFEPPASKLTWE

LVIKPMIGLGSTDDVIVKESEEQLSKVLDIYETRLTESKYLGGDSFTLVDLHHI

PNIYHLMNTKAKALFDSRPRVSVWCADILARPAWVKGLEKMQK

>SlGSTF5 Solyc12g094430.1.1

MATPVKVYGPTLSTAVSRVLACLLEKNVQFHLIPVNMAKGEHKKPAYLKIQ

PFGQVPAYQDEDITLFESRSINRYICDKYGSQGNKGLYGTNPLEKASIDQWI

EAEGQSFNPPSSVLVFQLAFAPRMKLKQDENLIRQNEEKLKKVLDVYEKRL

GDSQYLAGDEFTLADLSHLPNIQYLVNGTDRAELITSRENVGRWWGEISNR

ESWKKVVEMQTSPPPS

>SlGSTL1 Solyc04g009530.2.1

MAASSIGHQIHINVNSPILLPLRTNFSSLSFTFSNARYPLKWNHIGCPKICAL

PAVSIIASGSSREMLPPALDSSSEPPAIFDGTPKLYISYSCPYAQRTWIARNCK

ALQEEIKLVPIDLKNRPDWYKEKVYPANKVPSLEHNNEVKGESMDLIRYID

SNFEGPSLFPDDPSKREFAEELFSYFDSFYKAVISSLKEDKINDAIAAFDSIETA

LSKFVDGSFFLGSLSLVDIAYAPFIERFQPFLLEVKNYDITTGRTKLAAWIKEM

NQIEGYTVTKRDPKEHLENYKRRFLSQL

>SlGSTL2 Solyc09g007150.2.1

MAALSVQEVLPATLESTSEPPSLFDGTTRLYINYQCPYSQRVWITRNVKGLQ

DKINLVPIDLQNMPDWYKEKVYPQNKVPSLEHNNKMIGESLDLVKYVDSN

FEGPSLLPDDPEKRKFAEELIAYSDIFVPEVYKSFFRDAQTLAGAQFDYLEKA

LDKFDDGPFFLGQFSQVDIAYVPFIERFQIFMEKGINYDITSARPKLAKLIEE

MNKLDGYKQTKVLDPEKLVEYYKNLFLKKA

>SlGSTL3 Solyc10g084400.1.1

MATPSVQEIRPASLDSTSESPALFDGTTRLYISYVCPFAQRPWIARNFKGLQD

KIELVPIDLQNRPVWYKEKVYPQNKVPSLEHNNKVIGESLDLVKYIDSNFEG

PFLLPDDPEKQKFAEELIAYSDTFLKEIYANFKGDIEKHAGPQFDYLEKALD

KFDDGPFFLGQFSQVDIVYAPFVERFQIFLKEGLNYDITSGRPKLAKWTEEL

NKLDSYIQTKADPKEVVDLYKKKYLA

>SlGSTL4 Solyc12g044520.1.1

MASPSVQDLLPPSLDSTSQPPSLFDGTTRLYINYQCPYSQRVWITRNVKGLQ

DMIKLVPIDLQNRPDWYKENVYPKNKVPSLEHNNKVTGESLVLVKYVDCN

FEGPSFLPDDQEKRKFAEELIAYSDTTFVPEVYRSFAKDARTLAGAQFDYLE

KALHKFDDGPFFLGQFSQVDIIYAPFVERFHVFMPEGFNYDITTGRPKLAK

WTEEMNNLDGYKQTKVLEQEKMIEYYKNRFLPKA

>SlGSTL5 Solyc12g044530.1.1

MLLTQPVLLLSPLPFKKKQLSMASPSVQDLLPPSLDSTSQPPSLFDGTTRLYI

NYQCPYSQRVWITRNVKGLQDMIKLVPIDLQNRPDWYKEKVYPKNKVPSL

EHNNKVTGESLVLVKYVDCNFEGPSFLPDDQEKRKFVEELIAYSDTTFVPEV

YKSFAKDARTQAGVQFDYLEKALHKFDDGPFFLGQLSQVDIIYAPFVERFH

VFMPEGFNYDITTGRPKLAKWIEEMNNLDGYKQTKVLEQEKMVGYYKNR

FLLVPTS

>SlGSTT1 Solyc04g050560.2.1

MLDWHHSNLRHGTAGYIFDTVLAPAFGLTLNPQAAAEAEKVLLASLANIE

SVWIQKKGRLLLGSGQPSIADLSLVRELMELEAKHKHLKPRQRICSVN

>SlGSTT2 Solyc08g080900.2.1

MTLKLYVDRMSQPSRAVIIFCKLNGIDFEEIHINLSKRQQLSPEFKEINPMKQ

VPAIIDGRFKLFESHAILRYLACAFPGIADHWYPADLYKRAKVDSVLDWHH

SNLRRGAAGYIFNTVLAPAFGLPLNPQAAAEAEKVLLASLAKVESVWLQR

KGRFLLGSGQPSIADLSLVCEIMELEILDEKDRERIIGPYKRVLKWIDDTKNA

MEPHFQEVHVILFKAKEKFHKQRHAVGSSIPQSSRKPDLHSKM

>SlGSTT3 Solyc08g080910.2.1

MTLKLYVDRMSQACREVIIFCKLNGIDFEEVHIDLSKRQQLSPEYREINPIRQ

IPAIMDGRFKLSESHAILKYLACAFPRIADHWYPADLYKRAKVESVLDWHR

TNFPRGPGSYTFYSVLAPTVGLPLNTKAAARTEKMFIACLATIESVWLQKK

GRFLLGSDQPSIADLSLACEIMQLEILDEKDRERILGPFKRVLKWLDDTKNA

MAPHFEEVQSTLAGYKEKVQKQRNTLGSKITQSGRKPVLQSNM

>SlGSTT4 Solyc12g056250.1.1

MSLKVYVDRLSQPSRAILIFCKLNGIEFEEVNIDLAKGQHRTPEYQEVNIMK

QVPAIVHDTFKLFESHAILRYLASAFPETADHWYPKDLQKRANVECVLDW

HHANLRRGSAGYVFNTILAPAFGLPLNPQAAAEGKNLLSASLATIDTYWL

QKDGSFLLGNSQPSLADLSLVCEIMQLQFLDEKDREGLLSPHKNVLKWIDD

VKSATAPYFDEIHATLFKVSEIFQKQRAGGASS

>SlGSTZ1 Solyc01g091330.2.1

MKINPPMLLQISRSYIKLVKADNFQLWFGRHFSNTVTPTTNSIVDVSLAVPIS

GSSMESDNSMKKATDSTWVSKIVLYSFWQSSCSWRVRFALNLKGLSYEYRA

VNLGKGEQFTSEFDKLNPLHYVPVLVDGDVVISDSYAILLYLEEKYHQRPLL

PIKPQLRALNLQAASIVSSNMQPLHMLSVLRYMEERVGPEEKQLWAKFHIQ

KGFGALEKLLTGSAGKYATGDEVYMADVFLAPQIAVATKRFDIDMSEFPTL

RKIYDSCEALPEFQASLPERQPDASP

>SlGSTZ2 Solyc01g102660.2.1

MAGSGEESKKLQLYSYWRSSCAFRVRIALNLKGLDYEYKAVNLLKGEQRD

PEYLKLNPLGYVPTLVDGDAVIADSFAILMYLEEKYPQRALLPQDCQKRAI

NYQAANIVSANIQPLQNLAVLKYIQEKIGPDETTPWVQGHITKGFEALEKL
LKDYAGKYATGDEVYMADLFLAPQIHAAIKRFEVDMNQFPTLLRVFEAYQ
ELPAFQDAMPEKQPDAIHHL

>SlEF1Bg2 Solyc11g028090.1.1

MDLFLKAMLSHSTVRPCLIFFHLSSHHIAKYSFDTDFTNSRSYQINAQQSSF
GSSLFEYSEEAAVSALKRALGTLNTHLASTKYLVEHLITLADNIVVCNLSIGF
RMIMTKSFTKEIPRVERYFWTVVNQQNFSKILGKVKQAKSILAVQSKKPTQL
EKTKANEEPTKEVNKEEPSLVEKETTPKPKAKNPLDLLPPSKISG

>SlMGST Solyc02g081430.2.1

MAGVEFLPKEYGYVILALVVYCFFNFWMSFQVGKARKQYKVPYPTMYATE
AENKNANSFNCVQRGHQNSLEMMPTFFMLMIVGGIRHPLICASLGAVYIV
SRYFYFTGYSTGDPQNRLTLGKYNFLAIMALMICAASCGVNFLMS

>SlTCHQD Solyc04g057890.2.1

MQLYHHPFSLNSQKVRLTLEEKGIDYTSHHVNPLTGKNMDAFFFSMNPSA
KVPVFQNGSHIIYDTIEIIQYIERIAEKVSSGGNNLNLSSREVIGWMHKIQEW
DSMYFTLFHVPEKYRLCVSKFLRRVIIARMAESPDLASAYHCKLRQAYDTD
DKLKNADVLRRSENHLVRLLDEVELKLGETSYLAGEEFSLADVMLIPLLARI
ELLNLENEYINSRPNIADYWVLVKQRPSYKKVIGKYFDGWRRRKTLLKTW
CFIRVRSVLRKY
